# Association analysis of agronomic traits and construction of genetic networks by resequencing of 306 sugar beet (*Beta vulgaris* L*.*) lines

**DOI:** 10.1038/s41598-023-42182-2

**Published:** 2023-09-18

**Authors:** Liang Wang, Ziqiang Zhang, Pingan Han, Yahui Liang, Huizhong Zhang, Zengjuan Fu, Shangmin Zhao, Yuanyuan E, Hui Zhang, Xinrong Wu, Bizhou Zhang, Yue Chang, Kuangang Tang, Wenzhe Zheng, Li Chen, Ronghua Wang, Weishi Gao, Agula Hasi, Xiaodong Li, Chen Bai

**Affiliations:** 1 Key Laboratory of Herbage and Endemic Crop Biology, Ministry of Education, School of Life Sciences, Inner Mongolia University, Hohhot, China; 2 Key Laboratory of Sugar Beet Genetics and Germplasm Enhancement, Inner Mongolia Academy of Agricultural and Animal Husbandry Sciences, Hohhot, China; 3 College of Modern Agriculture and Ecological Environment, Heilongjiang University, Heilongjiang, China; 4 Beet Breeding and Seed Processing Laboratory, Institute for Sugar Beet Research, Shihezi Academy of Agricultural Sciences, Shihezi, China; 5 Research Industrial of Economic Crops, Xinjiang Academy of Agricultural Sciences, Urumuqi, China

**Keywords:** Plant breeding, Plant genetics, Plant molecular biology

## Abstract

Due to the relatively brief domestication history of sugar beet (*Beta vulgaris* ssp. *vulgaris*), our understanding of the genomic diversity and functional genes in its cultivars is limited, resulting in slow breeding progress. To address this issue, a total of 306 germplasm materials of major cultivars and breeding lines from China, the USA, and Europe were selected for genome resequencing. We investigated population structure and genetic diversity and performed selective scanning of genomic regions, identifying six novel genes associated with important agronomic traits: the candidate genes DFAX2 and P5CS for skin roughness; the candidate genes FRO5, GL24, and PPR91 for root yield and sugar yield, and the pleiotropic candidate gene POLX for flourishing growth vigour, plant height, crown size, flesh coarseness, and sugar yield. In addition, we constructed a protein–protein interaction network map and a phenotype-gene network map, which provide valuable information for identifying and characterizing functional genes affecting agronomic traits in sugar beet. Overall, our study sheds light on the future improvement of sugar beet agronomic traits at the molecular level.

## Introduction

Sugar beet (*Beta vulgaris* spp. *vulgaris* L.) is a core eudicot of the order Caryophyllales, family Amaranthaceae (formerly Chenopodiaceae), and subfamily Betoideae^1,2^. With an estimated genome size of 714–758 Mb, conventional sugar beet has 2n = 18 chromosomes^3^. It shares a common paleohexaploid ancestor with Rosaceae and Asteraceae^4^. Sugar beet is a major biennial root crop in temperate regions and is the world's second largest sugar crop after sugarcane. Global cane sugar production in 2021 was 130.964 mln tons, with sugar beet cultivation of 4,628,697 hectares producing 34.204 mln tons of sugar, or approximately 26% of global sugar production (International Sugar Organization Yearbook 2022). In addition, sugar beet is a source of bioethanol and animal feed^5^. Due to the abundance of wild resources and the continuous development of new varieties, the yield, quality and disease resistance of European and American sugar beet varieties have steadily improved. However, compared with foreign varieties, Chinese varieties have lower genetic diversity and limited breeding resources. Therefore, making good use of the existing core germplasm resources for pre-breeding and variety improvement is a priority for improving sugar beet yield, quality and disease resistance.

Beet has historically been grown as a vegetable or fodder crop, and the selection of sugar beet began at the end of the eighteenth century^6^. After long-term selection, its sugar content increased from 4% at that time to 6% and over 18% today^7^. Sugar beet in China started in the 1950s, and in the 1980s, many materials were introduced from Europe and the USA. These raw materials laid the foundation for breeding in China. Sugar beet cultivation in China is mainly distributed in the three ecotopes of Northeast, North and Northwest China. Breeders are most interested in the genetic diversity and population structure of the core resource materials in these regions as the starting material for improvement, as well as how to accurately mine the genes related to important agronomic traits.

Genome-wide association study (GWAS) is a genome-wide polymorphism assay for genetic variation that uses a natural population or a collection of different individuals to obtain genotypes, which are then statistically analysed against a phenotype to determine whether a genomic variant is associated with a trait of interest. GWAS has become a standard approach for deciphering genotype–phenotype associations in many species due to the development of rapid genotyping and next-generation sequencing technology^8^. GWAS has been successfully applied to various crops, including rice^9^, corn^10^, cotton^11^, tomato^12^, bean^13^, foxtail millet^14^, sorghum^15^. In 2014, the sugar beet genome was sequenced for the first time, marking a significant milestone in sugar beet breeding^5^. Recently, the sequential non-initial assembly of the EL10 genome and the IMA1 genome of pure sugar beet lines has been completed^16,17^. The chard (B. *v. vulgaris* var. *cicla*) genome sequence has been published^18^, and the assembled genomes of wild-derived beet accessions have become available^19,20^. However, our knowledge of the sugar beet genome and its functional genes associated with important agronomic traits is still limited compared to, for example, that for corn, soybean and rice. This is due to the complex genetic background and long reproductive period of sugar beet. Therefore, many sugar beet varieties need to be further investigated at the genomic level, especially for molecular traits associated with potential genetic networks for important agronomic traits such as root yield and sugar content.

In this study, the genetic diversity of core germplasm resources from four growing regions representing three major ecotopes in northern China was examined, and association analysis was conducted on important agronomic traits related to yield and quality. We resequenced 306 sugar beet accessions from four growing regions representing three major ecological zones in northern China and performed genotypic and phenotypic analyses. Our major objective was to explore the dynamics of genomic variation in response to population selection in sugar beet from the three major ecological regions of northern China. In parallel, we conducted a GWAS of 26 phenotypic traits in this species. The aim of this study was to identify and characterize multiple patterns and genes associated with essential agronomic traits. Our results provide important insights into the genome structure of sugar beet, including novel genes associated with important agronomic traits and genetic networks^21^. This information could serve as a valuable reference for molecular breeding and evolutionary studies in sugar beet, potentially supporting the development of new sugar beet varieties^22^.

## Results

### Genome resequencing approach for genotyping 306 sugar beet germplasm resources

In this study, we performed high-depth genome-wide resequencing of 306 sugar beet accessions using an Illumina HiSeq 2000 sequencer, obtaining 1977.12 Gb of sequencing data. This collection included 72 endemic accessions from Northeast China (Harbin), 114 endemic accessions from North China (Hohhot), 100 endemic accessions from Northwest China (50 from Urumqi and 50 from Shihezi), and 20 accessions from abroad. This diverse population represents the genetic diversity of ecotypes, geographical locations, and various traits. After filtering the raw sequencing data (see Materials and Methods), the high-quality clean data were aligned to the sugar beet reference genome, RefBeet-1.2.2 (http://plants.ensembl.org/Beta_vulgaris/Info/Index). The resulting high-density and high-quality genotype data covered 566,181,630 bp with an average sequencing depth of 11 × and a maximum depth of 22.5 × per accession. The average alignment rate for the population samples was 96.43% ± 0.5%, with an effective localization rate of 74.8%. The average sequencing depth across the genome (excluding gap regions) was 12.2 ×  ± 1.1 × . The genome coverage was 86% ± 1.8%. The high mapping and coverage rates ensured the reliability and high quality of the sequencing data. This comprehensive dataset allowed for a more accurate assessment of genetic diversity and identification of potential candidate genes associated with agronomic traits in sugar beet accessions.

A total of 18,875,282 variant loci were detected, which included 14,900,035 single nucleotide polymorphism (SNP) loci and 3,975,247 insertion/deletion (INDEL) loci. After screening for a gene frequency (minor allele frequency, MAF) greater than 0.05, 8,258,753 SNPs were identified. Among them, 872,623 were located 1 kb upstream of the genes, and 512,777 variants were in exonic regions, resulting in 242,816 non-synonymous mutations and 269,961 synonymous mutations. The ratio of non-synonymous SNPs to synonymous SNPs was 0.899. A total of 2,961,245 variants were found in intronic regions, 3309 variants were in splice sites, and 748,749 variants were upstream of one gene and downstream of another gene. Additionally, 12,439,140 variants were located in intergenic regions (Table 1).

**Table 1:** Single Nucleotide Polymorphisms (SNPs) statistics.

Category		Number of SNPs
Total		8,358,753
Indel		3,975,247
Upstream		872,623
Exonic	Non-synonymous	242,816
	Synonymous	269,961
Intronic		2,961,245
Splicing		3309
Downstream		748,749
Upstream/Downstream		67,984
Intergenic		12,439,140

### Population structure analysis of 306 sugar beet germplasm resources in different production areas

**Figure 1:**
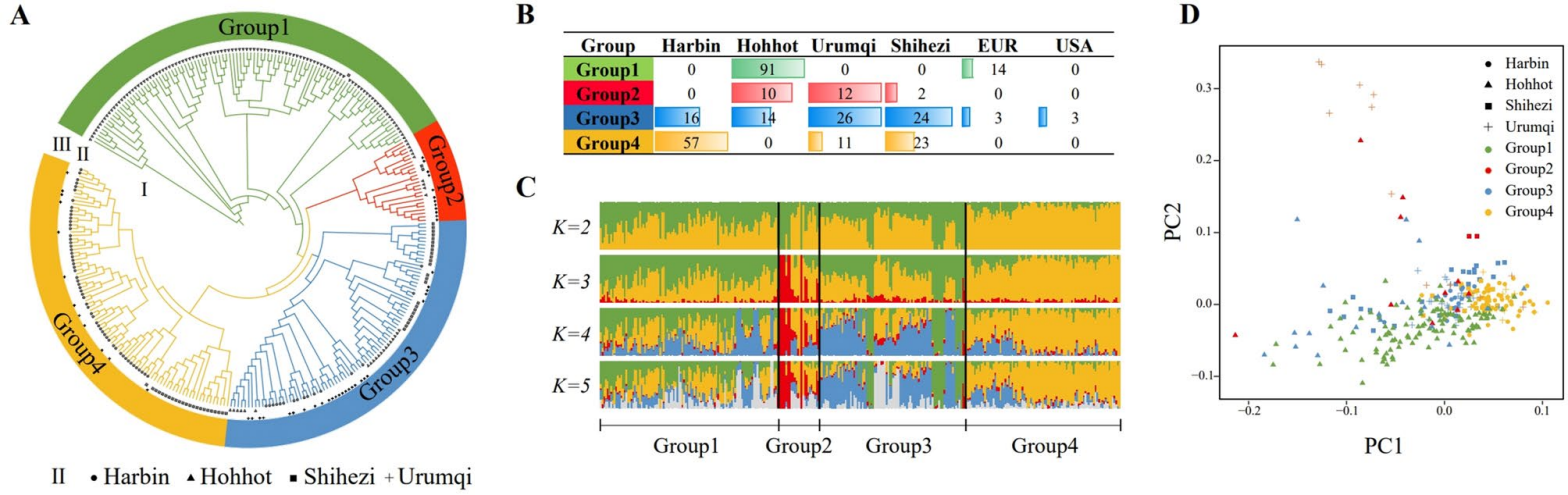
Population structure analysis of 306 sugar beet different subpopulations and different production areas.** (A)** Phylogenetic tree constructed for all sugar beet attributions. Groups 1–4 are indicated by different colors, and materials from different production areas are indicated by different shapes. (**B)** Production area source statistics for each subpopulation. (**C)** Mixed ancestry analysis for sugar beet subpopulations. Each color represents an ancestral component. K is set from 2 to 5 to track different ancestral components. (**D)** Principal component analysis of the first two eigenvectors for all sugar beet raw materials. Materials from different production areas are shown in different shapes, while subpopulations are shown in different colors.

We investigated the phylogenetic relationships among 306 germplasm resources by genome-wide SNP analysis. Phylogenetic trees were constructed for the resources from the four major production areas using the neighbor-joining (NJ) method, and the diversity of genotypes among these materials was assessed. The 306 accessions were divided into four groups based on the phylogenetic tree and genetic distance (Fig. 1A). The first group of 105 materials was dominated by introduced materials from Hohhot and Europe, with 91 from Hohhot and 14 from Europe. The second group, comprising 24 materials, consisted mostly of samples from Hohhot and Urumqi, with ten from Hohhot, 12 from Urumqi, and two from Shihezi. The third group included 86 materials, with 16 from Harbin, 14 from Hohhot, 26 from Urumqi, 24 from Shihezi, and three each from Europe and the United States. The fourth group, containing 91 materials, was mainly from Harbin but also included samples from Shihezi and Urumqi, with 57 from Harbin, 23 from Shihezi, and 11 from Urumqi (Fig. 1B).

We conducted a structure analysis on 306 sugar beet samples using Admixture software to gain insights into the genetic makeup and relationships of the germplasm resources. Each column in the image represents one individual, and the length of the differently coloured segments indicates the proportion of an ancestor in the individual's genome (Fig. 1C). When K = 2, the major ancestral component (yellow) of group 4 splits, indicating the highest level of selection in group 4. When K = 3, a new ancestral component (red) infiltrates, and two ancestral components, red and yellow, dominate in group 4. When K = 4, the optimal number of ancestral groups, we observe that the yellow ancestors of group 4 dominate, and the group has relatively homogeneous ancestors and a simpler genetic background compared to those of the other three groups. In contrast, the ancestral origins of group 1, group 2, and group 3 were relatively diverse. Yellow, green, and blue, representing different ancestral components, were relatively balanced in group 1, blue was relatively high in group 3, followed by green, and red and yellow ancestral components were dominant in group 2.

Principal component analysis (PCA) showed that some samples from group 2 were more clearly separated from the other three subgroups in the upper left corner, group 1 and group 3 both radiated to the lower left, group 4 samples were more centrally distributed, and only a few samples deviated from the other three subgroups and diverged to the lower right. With respect to the different production areas, samples from the Harbin region were tightly clustered. In contrast, samples from the Hohhot region showed the greatest dispersion, and the few samples from the Urumqi production area showed significant separation (Fig. 1D). Overall, however, the materials from the four regions did not show significant segregation and did not resemble a clearly differentiated population.

The above analysis showed that materials from different subpopulations and production areas had similar genetic backgrounds, indicating a high degree of gene exchange and interpenetration between resource materials within subpopulations. The analysis also indicates that since the introduction of sugar beet germplasm resources in China, some resource materials from the Hohhot area have been domesticated for many years, resulting in segregation from materials from other production areas, and materials from the Harbin area have a relatively homogeneous genetic background. This observation suggests that in China, sugar beet, as an exotic species, has a simple genetic background and low genetic diversity due to the lack of wild resources. After its introduction to China, sugar beet had a short reproductive period and underwent frequent genetic exchange between materials from different regions without forming independent subgroups with regional characteristics.

### Genetic diversity and selective scanning analysis

**Figure 2:**
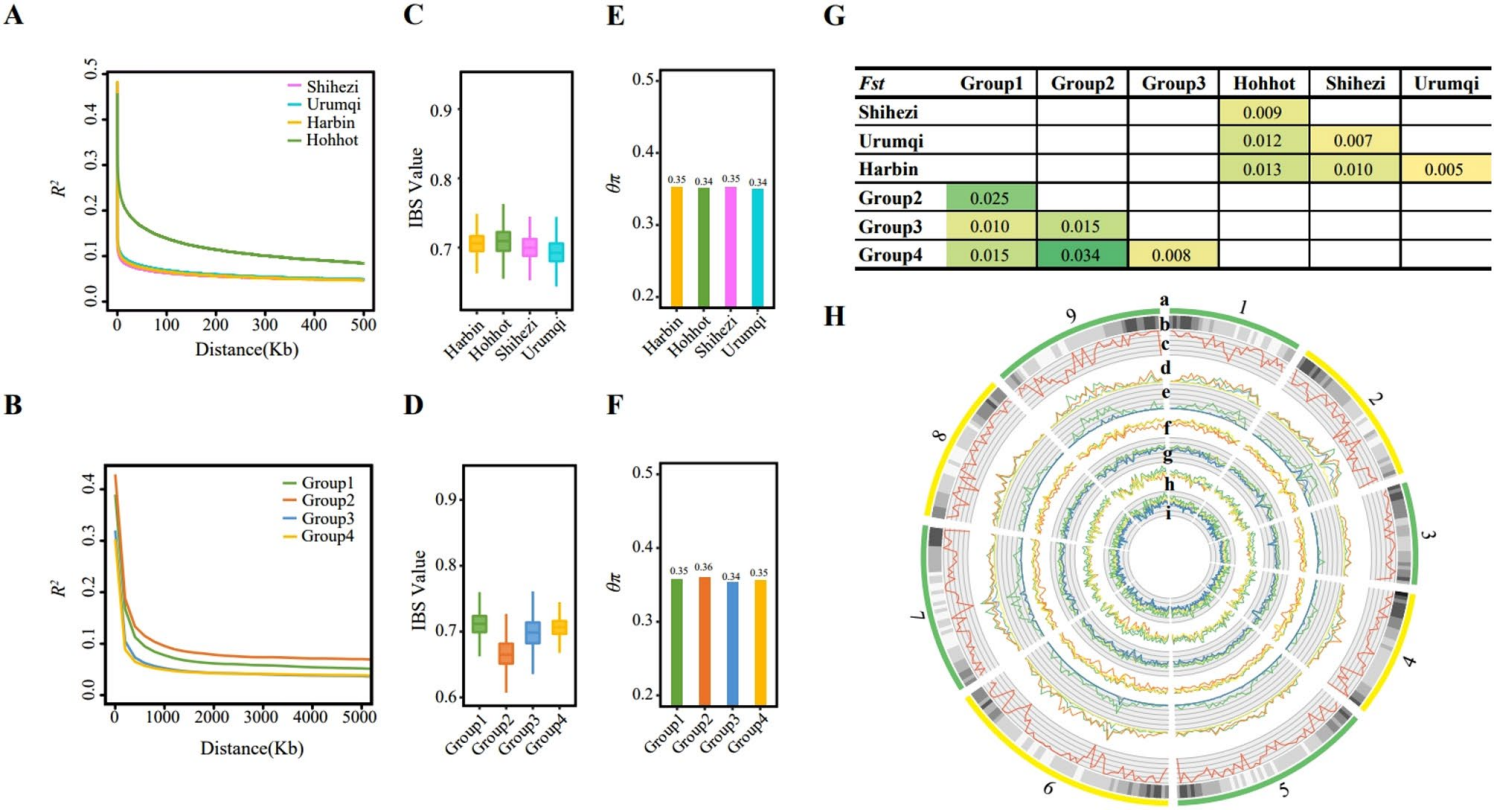
Genetic diversity analysis and putative selective regions of sugar beet resources from different subpopulations and production areas. **(A)** LD decay plots for sugar beet subpopulations. (**B)** LD decay plots for Harbin, Hohhot, Shihezi, Urumqi. (**C)** IBS value distribution for Harbin, Hohhot, Shihezi, Urumqi. (**D**) IBS value distribution for sugar beet subpopulations. (**E**) Comparison of θπ values for sugar beet subpopulations. (**F**) Comparison of θπ values for Harbin, Hohhot, Shihezi, Urumqi. (**G**) Comparison of Fst values between Harbin Hohhot Shihezi Urumqi. (**H**) Landscape of sugar beet genetic diversity across the whole genome. (a) Chromosomes. (b) Density of genes. (c) Density of SNPs (red). (d) LD value distribution for Group1 (green), Group2 (orange), Group3 (blue) and Group4 (yellow). (e) LD value distribution for Harbin (yellow), Hohhot (green), Shihezi (purple) and Urumqi (blue). (f) Tajima’s D value distribution for Group1 (green), Group2 (orange), Group3(blue) and Group4 (yellow). (g) Tajima’s D value distribution for Harbin (yellow), Hohhot (green), Shihezi (purple) and Urumqi (blue). (h) θπ value distribution for Group1 (green), Group2 (orange), Group3 (blue) and Group4 (yellow). (i) θπ value distribution for Harbin (yellow), Hohhot (green), Shihezi (purple) and Urumqi (blue). LD, linkage disequilibrium; IBS, identical-by-state; SNP, single-nucleotide polymorphism.

The results of linkage disequilibrium (LD) analysis showed that the LD decay distances of the four subpopulations were not significantly different, with G2 > G1 > G3 and G4 (Fig. 2A). Further analysis of the LD decay distances of the four production areas indicated that Hohhot had the largest distance, followed by Urumqi, Harbin, and Shihezi (Fig. 2B). These results suggest that the materials in the Hohhot region have experienced a higher degree of domestication and selection and greater selection intensity and have lower genetic diversity than those in the other three production areas. The results also show that the LD of materials in the Hohhot region is higher than that in the other three production areas.

The identity-by-state (IBS) analysis determines the concordance of all genetic markers to reflect the degree of correlation among individuals. This analysis revealed that group 2 was the smallest among the four subgroups, while groups 1, 3, and 4 were convergent (Fig. 2C). The average IBS values in the Hohhot production area were slightly higher than those in the other three production areas, which were very similar. Group 2 had the largest attenuated LD distance but the smallest mean IBS value (Fig. 2D). The decay distance of IBS values and LD show the same trend among the four regions.

Genetic diversity within a population can be measured using θπ value, which represents the number of different loci between any individuals within the population. This value is useful in understanding the genetic variation within a group and can provide information and guidance for breeding programs. The θπ values within the four subpopulations and clusters in the four production areas did not differ significantly, indicating that the genetic diversity among materials within different subpopulations and different production areas was similar (Fig. 2E, F).

The fixation index (Fst) was calculated to represent the genetic distance and differentiation between populations. The Fst values between the four subpopulations and the four production areas showed that G3 and G4 had the lowest values, with little differentiation and a close genetic distance. The Fst values of G2 and G4 were the highest, indicating the greatest degree of differentiation between these two subgroups (Fig. 2G). The Fst values were calculated among the four production areas. The highest Fst values were found between Harbin and Hohhot, indicating that the resource materials in these two areas were the most differentiated. Meanwhile, the Fst values between Hohhot and the other three production areas, compared to the values among the other three areas, all showed a significant degree of differentiation. Harbin and Urumqi had the lowest Fst values, suggesting that these two areas had the least differentiation, closest genetic distance, and most frequent gene exchange.

LD, Tajima's D, and Fst are effective metrics for studying genetic variation, and in this study, they were employed in pairs to investigate the genetic diversity of all materials, as shown in Fig. 2H, which illustrates the genome-wide genetic diversity of sugar beets.

### Phenotypically associated loci and genes identified using GWAS

We conducted a GWAS of 26 agronomic traits in 306 sugar beet accessions from four geographical locations (Harbin, Hohhot, Urumqi, and Shihezi) in three major ecological regions (Northeast, North, and Northwest China). Differences between traits were determined by calculating Pearson correlation coefficients of the traits (Supplementary Figure S1), root yield, sucrose content and sugar yield were also statistically analyzed (Supplementary Table 1). The results showed that root yield and sugar yield were positively correlated, with the highest correlation coefficient of 0.81. Plant height and flesh coarseness and plant height and root yield were positively correlated, with correlation coefficients greater than 0.5. Flourishing growth vigour and plant height, plant height and crown size, cotyledon leaf area and sucrose content, and seedling growth vigour and damping-off were negatively correlated, with correlation coefficients less than − 0.5.

**Figure 3:**
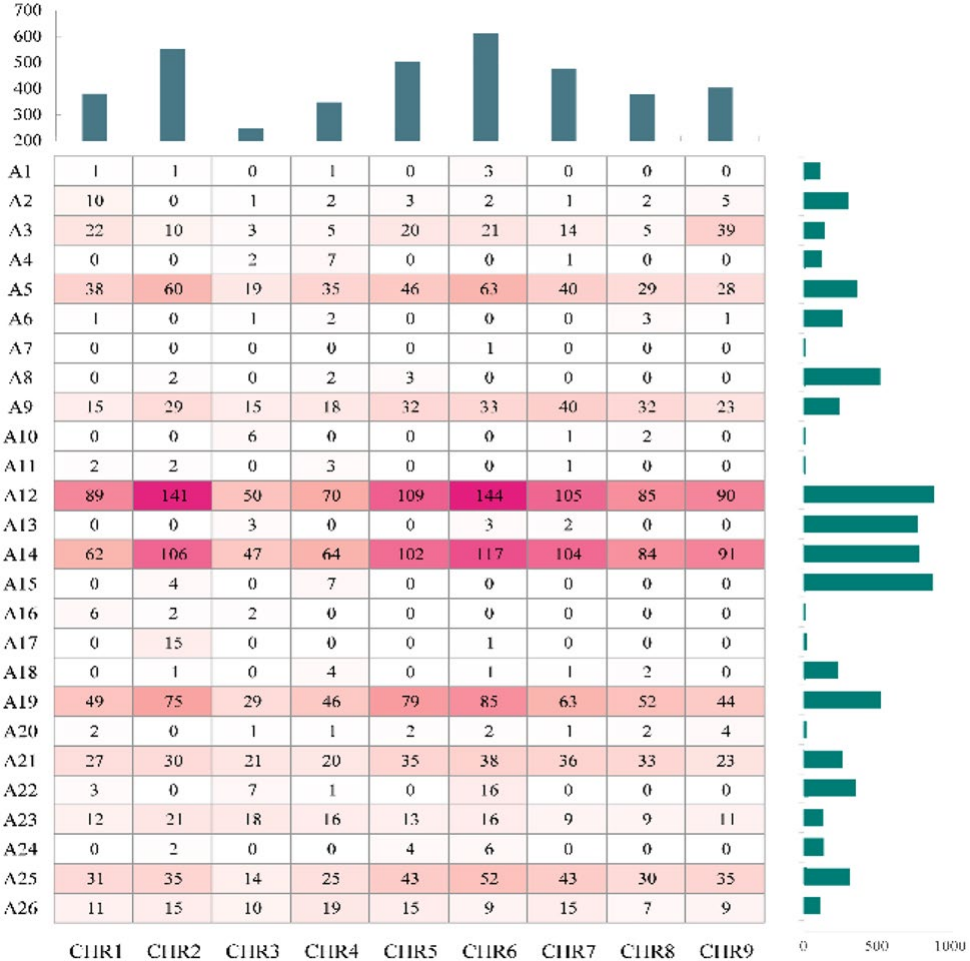
Distribution of genes associated with agronomic traits on different chromosomes.

We obtained 8,358,753 SNPs for subsequent analysis by screening with a gene frequency (MAF) greater than 0.05. The GWAS of the 26 agronomic traits was performed using Genomic Association and Prediction Integrated Tool (GAPIT) with the maximum likelihood method mixed linear model (MLM), and Manhattan and QQ plots were generated (Supplementary Figure S2–S27). In total, 3904 associated genes were identified, and the distribution of each trait on different chromosomes is shown in Fig. 3. The phenotype ID cross-reference table is shown in Table 2.

**Table 2:** Phenotype ID comparison table.

ID	Phenotype	ID	Phenotype
A1	Hypocotyl colour	A14	Crown size
A2	Leaf area of cotyledon	A15	Root groove depth
A3	Cotyledon size	A16	Skin colour
A4	Seedling growth vigour	A17	Skin roughness
A5	Flourishing growth vigour	A18	Flesh colour
A6	Leaf colour	A19	Flesh coarseness
A7	Surface shape of leaf	A20	Ring number of bundle
A8	Margin shape of leaf	A21	Root yield
A9	Leaf shape	A22	Sugar yield
A10	Petiole length	A23	Sucrose content
A11	Fascicled	A24	Damping-off
A12	Plant height	A25	Root rot
A13	Root shape	A26	Economic type

### Genes associated with skin roughness

**Figure 4:**
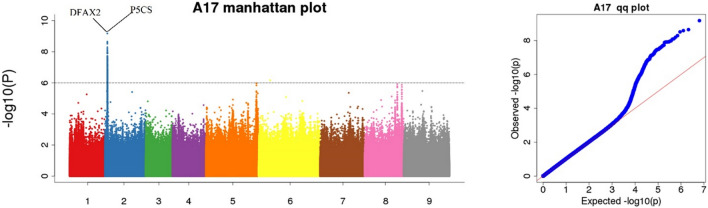
Manhattan plot and QQ plot of candidate genes related to the sugar beet skin roughness.

Primary roots with a smooth epidermis reduce soil carryover at harvest time, and the smooth epidermis also acts as a barrier preventing bacteria and viruses from invading the primary roots^23^. In this study, a total of 599 SNP loci associated with skin roughness were identified, with 16 loci being associated (Fig. 4) and 14 loci ultimately annotated. The two genes with the strongest association signals among the 14 annotated genes were DFAX2 and P5CS (Table 3), with the highest − log10(P) of 9.17703. Both genes are located on chromosome 2 and share a common mutation site, 2,312,199, in the intergenic region. Gene annotation revealed that DFAX2 is involved in the formation of the defensin-like protein AX2 and P5CS is involved in the formation of δ-1-pyrroline-5-carboxylate synthase. In subsequent qRT-PCR validation, the expression of DFAX2 was significantly higher in the unsmooth-epidermis material than in the smooth-epidermis material. The expression of P5CS also showed a significant change.

**Table 3:** Functional variation of genes related to skin roughness.

Chromosome	Position	Number SNP	Peak value	Reference	Alternative	Type	Gene ID	Gene name
2	2312199	78	9.17703	A	T	intergenic	BVRB_2g025520	DFAX2
2	2312199	78	9.17703	A	T	intergenic	BVRB_2g025540	P5CS

### Genes associated with sugar yield

**Figure 5:**
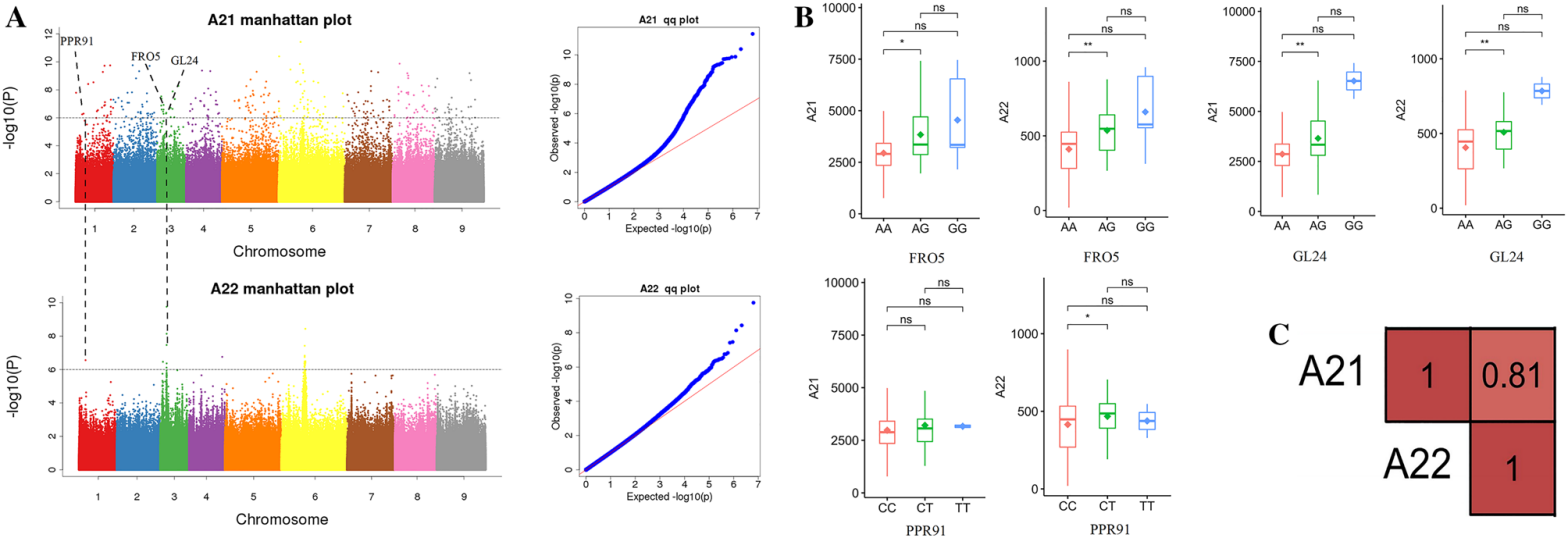
Three genes related to the sugar beet root yield and sugar yield identified by GWAS. **(A)** Manhattan plot of root yield and sugar yield and the candidate FRO5, GL24, PPR91 genes. (**B)** The two traits associated with sugar yield distribution for the haplotypes of PPR91, GL24, and FRO5. *P < 0.05; **P < 0.01; ns, not significant. (**C)** Phenotype correlation of root yield and sugar yield relate traits.

Sugar yield is a critical trait for sugar beet growers, as it is calculated based on the weight of roots per hectare and the percentage of root sucrose (°S)^24^. Previous studies on sugar beet consistently demonstrated a strong negative correlation between sucrose content and root yield. Several factors in sugar beet can affect yield (e.g., mass/area) and physiological components (e.g., the proportion of total mass). Due to this characteristic of sugar beet, the present study introduces the complex trait of sugar yield as a means to investigate yield quality. For the sugar yield trait, a total of 10 candidate genes were identified. After further analysis, three genes, FRO5 (BVRB_3g053570), GL24 (BVRB_3g053550), and PPR91 (BVRB_1g005550), two on chromosome 3 and one on chromosome 1, were found to be associated with both root yield and sugar yield (Fig. 5A, Table 4). Gene FRO5 mutations were located downstream, GL24 mutations in the intergenic region, and PPR91 mutations in the UTR5. Gene annotation revealed that FRO5 is involved in regulating iron reduction via oxidase, GL24 is a member of the sprouting protein subfamily 2, and PPR91 is involved in the construction of At1g62670, a protein containing pentapeptide repeats in mitochondria. The results show that functional mutations of the genes FRO5 and GL24 yield three haplotypes, AA, AG, and GG, for each gene. GG mutations were primarily concentrated in the breeding materials of Urumqi and belonged to the subgroup materials of group 4. Correlation analysis by phenotype showed that the correlation coefficient between root yield and sugar yield was 0.81 (Fig. 5C), indicating a significant positive correlation. In haplotype comparisons, both AA and AG haplotypes showed significant or highly significant correlations. Functional PPR91 gene mutations, i.e., CC and CT, showed a significant correlation (Fig. 5B). Following qRT-PCR validation, the expression of two genes, FRO5 and GL24, was found to decrease with decreasing root yield and sugar yield, whereas the expression of the PPR91 gene increased considerably with decreasing root yield and sugar yield.

**Table 4:** Functional variation of genes related to root yield and sugar yield.

Chromosome	Position	Number SNP	Reference	Alternative	Type	Gene ID	Gene Name
3	5850027	3	A	G	downstream	BVRB**_**3g053570	FRO5
3	5825259	2	A	G	intergenic	BVRB_3g053550	GL24
1	6091169	1	C	T	UTR5	BVRB_1g005550	PPR91

### POLX was associated with sugar yield

**Figure 6:**
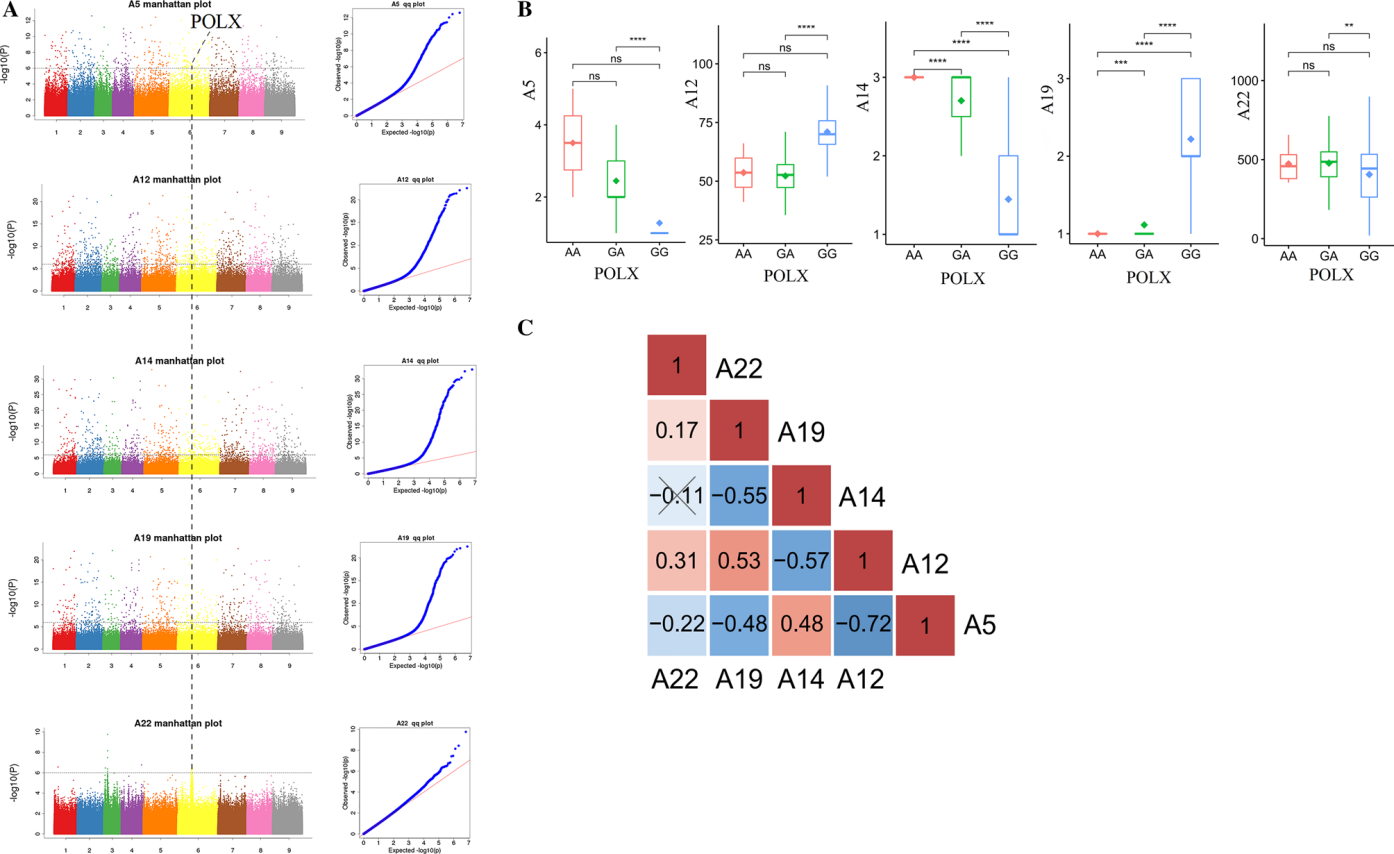
POLX was associated with sugar yield traits.** (A)** Manhattan plot and qq plot of candidate genes related to the sugar flourshing growth vigour, plant height, crown size, flesh coarseness and sugar yield. (**B)** The five traits associated with sugar yield distribution for the haplotypes of POLX. **P < 0.01; ***P < 0.001; ****P < 0.0001; ns, not significant. (**C)** Phenotype correlation of five traits associated with sugar yield.

The five traits associated with sugar yield (flourishing growth vigour, plant height, crown size, flesh coarseness and sugar yield) shared a common gene, POLX (BVRB_1g140620), located on chromosome 6 (Fig. 6A). Mutations occurred at two positions, 21,650,496 and 21,669,194, from G to A (Supplementary Table 2). The relationship between the two functional mutations was further analysed, with four mutations located upstream and one in the intronic region (Table 5). Phenotypic correlations were observed: flourishing growth vigour and crown size, plant height and flesh coarseness, plant height and sugar yield, and flesh coarseness and sugar yield showed a positive correlation, while flourishing growth vigour and plant height, flourishing growth vigour and flesh coarseness, plant height and crown size, and crown size and flesh coarseness exhibit a negative correlation (Fig. 6C).

**Table 5:** Functional variation of genes related to flourishing growth vigour, plant height, crown size, flesh coarseness, and sugar yield.

Chromosome	Position	Number SNP	Reference	Alternative	Type	Gene ID	Gene Name
6	21650496	4	G	A	upstream	BVRB**_**6g140620	POLX

The results reveal that the functional mutations formed three haplotypes, AA, GA, and GG, for each gene. Comparisons of haplotypes and phenotypes separately showed that for flourishing growth vigour and plant height, the phenotypes of GA and GG displayed a highly significant correlation; for crown size and flesh coarseness, AA, GA, and GG all exhibited a highly significant correlation; and for sugar yield, the phenotypes of GA and GG exhibited a significant correlation (Fig. 6B). Following qRT-PCR validation, the results showed that the expression of POLX changed significantly with different haplotype phenotypic changes.

### Construction phenotype-gene networks using central gene modules and multiple genes

**Figure 7:**
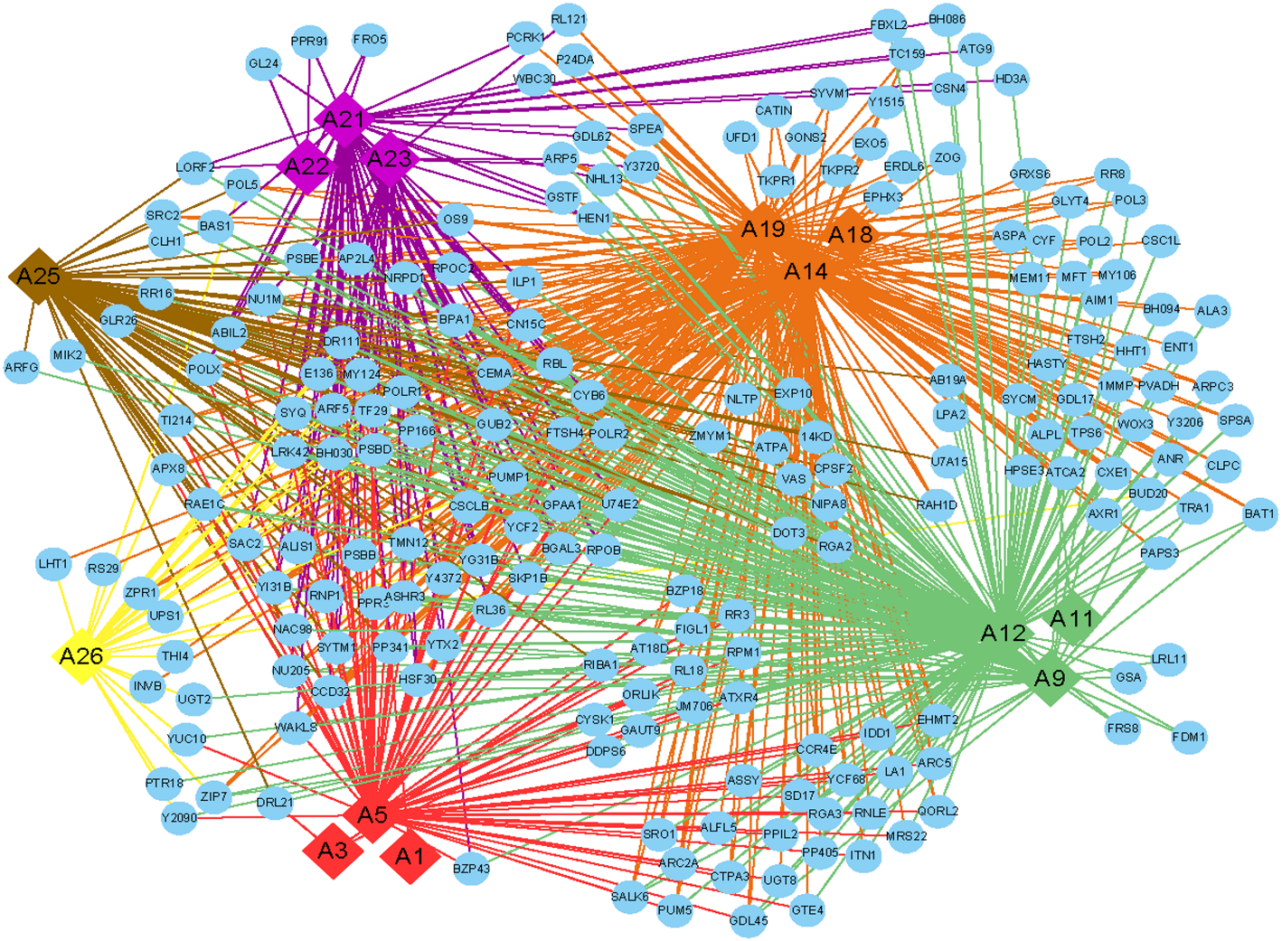
Phenotype-gene association/interaction network for fourteen trait categories in sugar beet. Traits are represented as solid rhombuses, and genes as solid circles.

**Figure 8:**
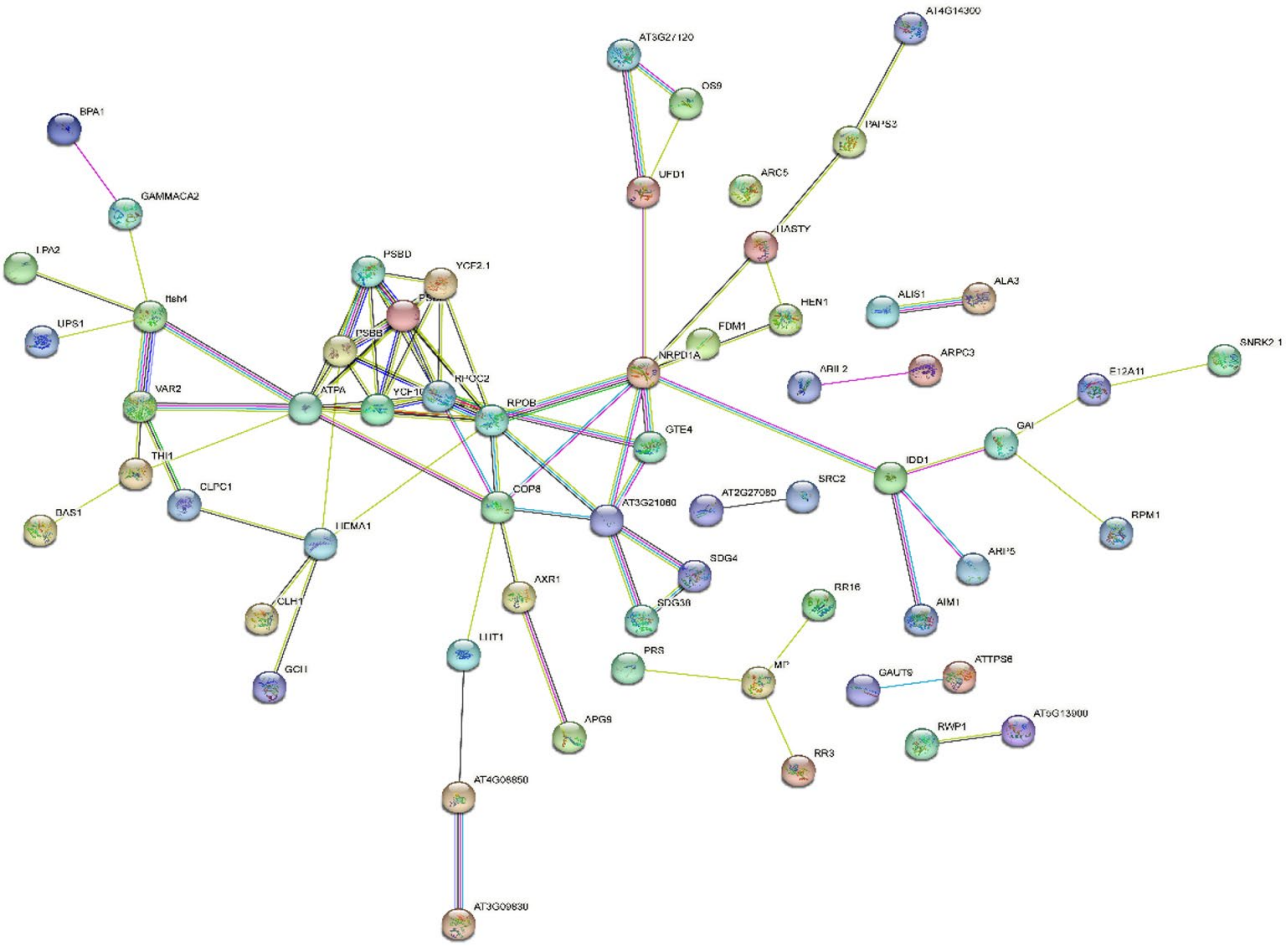
PPI gene network map of 14 traits and 256 annotated pleiotropic genes in sugar beet**.**

Through an in-depth exploration of the GWAS results, we identified instances where one trait was associated with multiple genes, and one gene was linked to multiple traits. Furthermore, we discovered complex networks between various phenotypes and genes due to extensive protein-level interactions among genes. To gain further insights, we performed a functional mutation-based haplotype test to construct a protein–protein interaction (PPI) network map and a phenotype-gene network map, which included 14 traits and 256 annotated pleiotropic genes (Figs. 7, 8). We categorized these traits into six categories: seedling traits, morphological traits, root traits, yield quality traits, root rot resistance, and economic type (Supplementary Table 3). Traits within the same category showed close links throughout the network.

In this genetic network, the six trait categories were linked by 14 central nodes containing 256 genes, with the largest central node being a root trait. In the PPI network diagram, the genetic network was dominated by ten major genes that linked different pleiotropic genes. The gene PPI network diagram allowed the identification of the pleiotropic genes of the major nodes. The combination of phenotypic associations and protein interactions with these primary genes provided a basis for identifying important candidate genes. This comprehensive information could be utilized to develop targeted breeding strategies, enhance the efficiency of genetic selection, and accelerate the process of cultivar development.

## Discussion

In this study, we conducted the first resequencing of 306 sugar beet germplasm accessions, providing valuable insights and supplementary data for sugar beet genome research. The selected materials in this study encompass resource materials from four major production areas in China. We performed the first-ever analysis of the genetic structure of germplasm materials from these critical production areas using population genetics and GWAS, determining the genetic diversity levels and differentiation degree among them. Our results demonstrated that despite different regions and breeders selecting the same materials through years of crossbreeding, these materials exhibited varying degrees of domestication while still maintaining similar genetic backgrounds.

We assessed the population classification of the resource material and examined their relationships with the domestication levels in other regions. Our comprehensive analysis incorporated a phylogenetic tree, PCA, and LD analysis. Our results showed that, in contrast to those from the other three production areas, the materials from the Hohhot region exhibited a high degree of domestication, a high level of differentiation, and reduced genetic diversity. We observed a trend where varieties with similar domestication levels tended to exhibit greater genetic similarity. However, varieties with similar genetic backgrounds still displayed differences in geographic distribution and domestication levels, implying that gene exchange might have occurred between germplasm of different populations. This phenomenon reflects the domestication history of sugar beet resource material in the four major planting regions after their introduction to China and highlights the increasing complexity of the sugar beet genetic background due to years of hybrid selection. Population structure analysis revealed a simpler genetic background in the northeastern region than in the other regions, suggesting that the resource materials derived from this area had a more homogeneous ancestral origin.

IBS, θπ, and Fst analyses revealed relatively consistent genetic diversity levels among individuals within different subpopulations and production areas. However, a higher degree of correlation was observed among individuals from the North China production area, suggesting that the germplasm resource materials from this region experienced multiple recombination events among individuals. Fst analysis indicated that the resource materials from Northeast and North China were the most differentiated. The genetic proximity and the lowest degree of differentiation suggest that the resource materials from the Northeast and Northwest production areas, particularly Harbin and Urumqi, have experienced more frequent genetic exchanges with each other.

This research revealed pleiotropic genes associated with skin roughness and sugar yield, which can be used to screen new candidate genes for future sugar beet breeding programmes. This information is very helpful for breeders to develop improved sugar beet varieties with desired traits, ultimately enhancing crop performance and overall production efficiency (Relevant SNP information was provided in Supplementary Table 4).

Root morphological traits are crucial for processing quality, harvesting operations and overall plant performance. Specifically, hybrids with smooth primary roots, i.e., primary roots without two vertical grooves and featuring a smooth epidermis, exhibit reduced soil carryover, which can otherwise cause damage during the slicing and spreading stages^25^. Researchers have developed smoother-rooted varieties with improved root shape and reduced crown size through repeated mass selection cycles, using hybrids of sugar beet and garden sugar beet^26,27^. Two potential genes, DAFX2 and P5CS, have been identified as strongly associated with the smooth epidermis trait. According to gene function annotation, DFAX2 encodes a defensin-like protein AX2, while P5CS encodes a delta-1-pyrroline-5-carboxylate synthase. These genes may play a significant role in determining root smoothness and overall sugar beet quality.

As the use of natural pathogen peptide preparations in agriculture and medicine has increased, plant-produced defensins have garnered more attention from researchers. Plant defensins are alkaline cysteine-rich proteins composed of 45–54 amino acids^28^. In this study, it was found that AX2 in sugar beet belonged to the "morphogenetic" plant defensins, which can not only inhibit the elongation of fungal hyphae but also inhibit the growth of bacteria^29^. This inhibition is achieved through Ca^2+^ signal transduction and destruction of the cytoplasmic Ca^2+^ gradient^30^.

Plants, including halophytes, often accumulate proline to cope with osmotic stress caused by high salinity and water shortage^31^. Some researchers have suggested that proline, similar to glycine betaine, acts as an osmotic protector to help plants resist changes in osmotic imbalance^32^. The expression of P5CS and P5CR genes in Arabidopsis and their relationship with proline accumulation under osmotic stress were studied. The results showed that P5CS gene induction preceded proline accumulation, indicating that P5CS plays a crucial role in proline biosynthesis under osmotic stress^33^.

The integrity of root bark is important in reducing bacterial and fungal infections and in limiting osmotic stress. Therefore, it is hypothesized that the two genes DAFX2 and P5CS, which are highly correlated with the smoothness of the root epidermis, play a role in protecting and repairing beetroot bark during antibacterial and anti-osmotic stress. However, further investigation is needed to verify this function.

In the association analysis of genes related to sugar production, we identified three strongly related genes. The first candidate gene was FRO5, which is related to copper (Cu) absorption in roots. Cu is an essential trace element required for various biological processes, and it has been selected as a cofactor of various protein active centres over the course of evolution due to its unique chemical properties^34^. The minimum concentration of Cu required by plants is approximately 5 μg g^−1^ leaf dry biomass, and if the concentration exceeds 20 μg g^−1^ dry biomass, Cu poisoning may occur^35^. Under the condition of Cu deficiency, typical symptoms of wild-type Cu deficiency can include chlorosis and a large reduction in aboveground and root biomass^36^. Thus, excessive Cu can lead to Cu poisoning, while Cu deficiency can cause various growth and yield reduction symptoms, such as chlorosis and biomass reduction.

A study of Arabidopsis spl7 mutants revealed that FRO5 and FRO4 transcription levels increased significantly in an spl7-dependent manner when Cu was deficient^37^. Further biochemical analysis and confocal imaging of Cu-specific fluorophores showed that FRO5 and FRO4 were involved in the absorption of high-affinity root Cu, specifically reducing Cu(I) by Cu(II), which caused a defect in iron transport from root to stem related to the lack of iron oxidase activity. These findings provide insight into the roles of FRO5 and FRO4 in regulating Cu and iron uptake in plants and suggest their potential use as targets for improving crop growth and yield.

The second gene was GL24, which encodes germin-like protein subfamily-2 member-4. Germin-like proteins (GLPs) are ubiquitous plant proteins that have been implicated in various biological processes, including the cold resistance response in rice^38^.

Finally, we identified the PPR91 candidate gene in our study, a member of the pentatricopeptide repeat (PPR) protein family, one of the largest and most complex protein families in plants. PPR proteins have been found to be involved in various crucial functions related to organelle biogenesis and plant development^39^. PPR-related mutants have been observed in several plant species, such as Chlamydomonas^40^. Arabidopsis^41^, maize^42^, turnip^43^, and Sorghum^44^. Further investigation is needed to determine the exact role of PPR91 in sugar beet and its potential application in breeding for improved sugar production.

The PPR protein family is characterized by a degenerate 35-amino acid repeat sequence, with each peptide arranged in a series with 2–27 repeats. Many PPR proteins are predicted to target either mitochondria or chloroplasts^45^. The PPR protein family plays an essential role in regulating gene expression related to cytoplasmic male sterility (CMS)^46^, participates in the regulation of embryo formation^47^, and influences plant growth and development^48^. In our study, we found a strong correlation between the candidate gene PPR91 and sugar production. Further investigation is needed to explore the precise mechanism by which this gene regulates sugar production in sugar beet plants.

We also identified a gene, POLX, among the genes related to sugar yield that was associated with multiple traits. POLX is annotated as a retrovirus-related Pol polyprotein from transposon TNT 1–94. Canto-Pastor et al*.* reported that disease resistance protein (TIR-NBS-LRR class), retrovirus-related Pol polyprotein from transposon TNT 1–94, and transposon Ty3-1 Gag-Pol polyprotein were targets of miRNA482/2118 members, and they could affect the expression of nucleotide binding site leucine-rich repeat (NLR) mRNAs and disease resistance^49^. The involvement of POLX in multiple traits suggests a potential role in regulating disease resistance and yield in sugar beet. In summary, the annotated functions of the genes related to sugar production are associated with stress resistance and disease resistance.

To grow and reproduce, many pathogens have evolved mechanisms to obtain glucose by hijacking the host's sugar outflow system. As a result, pathogenic bacteria can alter the sugar efflux of infected sites and modulate plant immunity^50^. Plants have developed sensing and response mechanisms to adapt to adverse environmental stresses such as drought, high salinity, and low temperature. As an osmotic protector and molecular switch, sugar regulates the resistance and adaptability of plants to stress^51^. It is hypothesized that the identified genes not only participate in the regulation of disease resistance and stress resistance but also play a role in regulating sugar production.

The primary objective of plant breeding is to combine several desirable traits into a single genome, which requires breeders to enhance and select multiple interdependent traits simultaneously^52^. However, selecting for favourable traits could inadvertently lead to the selection of undesirable traits since multiple traits can be interrelated. Understanding the genetic networks underlying different traits could benefit breeders in terms of enhancing breeding efficiency by helping them identify similarities among various genetic resources. For example, in recent decades, functional genomics in rice has rapidly developed, and some key genes controlling yield and grain quality have been identified^53^. Such efforts have enabled breeders to select and improve multiple related traits simultaneously, which is the ultimate goal of plant breeding.

In this study, we established a novel phenotypic-gene network that includes 14 traits and 247 annotated genes. The establishment of a phenotypic-gene network, as achieved here, can provide a more direct understanding of the relationships between genes and phenotypes, facilitating the discovery of important candidate genes for improving yield quality traits in sugar beet at the molecular level. By identifying these key genes, breeders can more precisely select desired traits while avoiding the selection of unwanted traits, ultimately leading to the development of more productive and sustainable crops.

## Materials and methods

### Plant materials and phenotyping analysis

The experimental materials for this study were provided by the College of Modern Agriculture and Ecology of Heilongjiang University, the Institute of Specialty Crops of the Inner Mongolia Autonomous Region Academy of Agricultural and Animal Husbandry Sciences, the Sugar Beet Research Institute of the Shihezi Academy of Agricultural Sciences, and the Economic Crop Industry Research Center of the Xinjiang Provincial Academy of Agricultural Sciences. The 306 materials were planted in four different locations with different environmental conditions in Northeast, North and Northwest China for phenotypic data collection. Seventy-two materials were planted at the experimental site in Hulan District, Harbin, Heilongjiang Province, Northeast China; 134 materials were planted at the experimental site in Hohhot (114 materials from Hohhot, 20 materials from foreign introduced varieties), Inner Mongolia Autonomous Region, North China; 50 materials were planted at the experimental site in Urumqi, Xinjiang Province, Northwest Region and 50 materials were planted at the experimental site in Shihezi (Supplementary Table 5). Phenotypic trait data were collected using quantitative and descriptive methods based on descriptors and data standards published by the National Crop Germplasm Resource Platform and the National Crop Science Data Center (http://www.cgris.net). Phenotypic correlation analysis using the R environment (gWQS package) was performed on the collected phenotypic data. Seeds were planted in pots and grown in a climate chamber (Percival USA) at 25 ± 1 °C in day/20 ± 1 °C in night (14 h day/10 h night) with 70% relative humidity until the 6-true leaf stage (approximately 4 weeks old). Seedling leaves were used for qRT-PCR validation.

### DNA preparation and sequencing

To prepare for DNA extraction, sugar beet leaves were collected from experimental plots in four growing regions. Leaves from a single plant per accession were harvested at the unifoliate growth stage, immediately frozen in liquid nitrogen, and stored at − 80 °C until processing. During grinding with a porcelain mortar and pestle, liquid nitrogen was added as needed to prevent thawing and replenished as it evaporated. Genomic DNA was extracted from the young leaves of each genotype using Plant Genomic DNA Kits (Tiangen, Beijing) according to the manufacturer's protocol. For each landrace, a single individual was used for genome sequencing on the Illumina HiSeq PE150 platform. Library construction and sample indexing were performed as described.

### Total RNA extraction, cDNA synthesis, and qRT-PCR analysis

Total RNA was isolated from each sample using polysaccharide polyphenol plant TRIzol Universal Extraction Reagent (Tiangen, Beijing, China) according to the manufacturer’s protocols. The purified RNA was stored at − 80 °C until further analysis. First-strand cDNA synthesis was performed using 5X Evo M-MLV RT Reaction Mix reverse transcriptase according to the manufacturer’s protocols (Accurate Biology, Hunan, China). qRT-PCR was performed using 2 × SG Green qPCR Mix (Vazyme, Nanjing, China) and a real-time PCR machine (ABI No. QuantStudio 7 Flex USA) according to the manufacturer’s protocols. The primer sequences used in the qRT-PCR were shown in Supplementary Table 6 The programme used for qRT-PCR was 95 °C for 3 min, followed by 40 cycles of 10 s at 95 °C and 30 s at 60 °C. Actin was used as a reference gene to analyse the relative expression patterns of mRNA. The reactions were performed with three biological replicates, with at least two technical replicates for each sample. The 2-∆∆Ct method was used to calculate the relative gene expression levels^54^. Finally, data from three biological replicates are presented as mean ± standard deviation (SD). The result was shown in Supplementary Table 7.

### Sequence alignment

The sugar beet reference genome (RefBeet-1.2.2) was used to align paired-end reads with Burrows-Wheeler Aligner^55^ (version: 0.7.8). This was carried out using the command line "BWA mem -t 4 -k 32 -M". After alignment, the sorted reads were run through the 'rmdup' program to remove any PCR duplicates. At this stage, only read pairs with the best mapping quality and identical external coordinates were retained.

### Population SNP detection

After alignment, we applied the Bayesian approach to SNP calling at the population level using the Samtools software^56^. Allele frequencies in the sample were calculated using a Bayesian method, and genotype likelihoods were determined based on the readings for each individual at each genomic site. We used the parameters '-q 1 -C 50 -S -D -m 2 -F 0.002 –u' with the 'mpileup' command to detect SNPs. To eliminate SNP calling errors caused by incorrect mapping or INDEls, only high-quality SNPs (coverage depth between 84.2 and 87.8, RMS mapping quality ≥ 20, and MAF ≥ 0.05) were retained for further analysis. After filtering, 8,258,753 SNPs were retained from the initial set of 18,875,282 raw SNPs.

### Functional annotation of genetic variants

SNP annotation was performed using the ANNOVAR package (Version: 2013-05-20) with alignment to the sugar beet DNA reference genome (RefBeet-1.2.2)^57^. Genome annotation was used to classify SNPs into different categories, including exonic (overlapping with a coding exon), intronic (overlapping with an intron), splicing site (within 2 bp of a splicing junction), upstream (within 1 kb of the transcription start site), downstream (within 1 kb of the transcription stop site), and intergenic regions. SNPs in coding exons were further categorized as either synonymous (no amino acid change) or non-synonymous (amino acid change). In this case, only the highest-quality SNPs were annotated. In addition, mutations causing stop gain (premature stop codon resulting in a truncated protein) and stop loss (removal or alteration of a natural stop codon resulting in an elongated protein) were also classified in this group.

### Phylogenetic tree and population structure

To gain deeper insights into the phylogenetic relationship from a genome-wide perspective, we constructed an individual-based NJ tree using the p-distance metric. This approach allowed us to investigate the genetic relatedness among the sugar beet accessions. We used TreeBeST software (http://treesoft.sourceforge.net/treebest.shtml) for tree construction and MEGA6.0 software (http://www.megasoftware.net/) for visualization of the phylogenetic trees, which revealed the clustering patterns and underlying genetic relationships among different sugar beet accessions. To further assess the genetic structure and identify possible population subgroups among the accessions, we performed PCA using GCTA software (http://cnsgenomics.com/software/gcta/mlmassoc.html). The Tracey-Widom test was used to analyse the eigenvectors' significance level, providing statistical support for the observed patterns of genetic variation. Using fastSTRUCTURE^58^, a population admixture analysis with K = 2 to K = 5 was performed to infer ancestral admixture.

### Genetic diversity analysis

LD analyses for each subpopulation were performed using PLINK by calculating the correlation coefficient (r^2^) of each SNP pair in a chromosome^59^. An LD decay plot was generated using the average r^2^ value for distances from 0 to 1000 kb. Pairwise IBS calculations were also performed using PLINK, and a distance matrix was generated for each subpopulation. Population genetic diversities were measured using VCFtools^60^ by calculating θπ and Fst. θπ was used to measure the genetic diversity of each subpopulation, while Fst was used to measure the genetic diversity between subpopulations. In addition, sliding window calculations of r^2^, θπ, and Fst were performed for genome-wide representations of sugar beet genetic diversity with a 100 kb window with 10 kb steps.

### GWAS

Association analysis between target traits and genome-wide SNPs across the subpopulations from structure analysis was carried out by using the GAPIT package in R software^61^. To minimize false positives and increase statistical power, an MLM analysis was performed by GAPIT without compression. The significance threshold of SNP-trait associations was established with a false-detection rate-adjusted P < 1 × 10^–6^ using the Benjamini–Hochberg procedure^62^. The GWAS results were displayed using a Manhattan plot and a QQ plot created with the R package CMplot^63^.

### Network construction

The most significant pleiotropic genes with P < 1 × 10^–6^ and their associated traits were retained among all localized and annotated pleiotropic genes to build the phenotype-gene network for sugar beet. PPI information for sugar beet was retrieved from the STRING database^64^ and mapped to the sugar beet genes using BLAST^65^. The construction, visualization, and exploration of the network were performed using Cytoscape^66^.

### Supplementary Information

Supplementary Information.

## Data Availability

The raw sequence data reported in this paper have been deposited in the Genome Sequence Archive (Genomics, Proteomics & Bioinformatics 2021) in National Genomics Data Center (Nucleic Acids Res 2022), China National Center for Bioinformation / Beijing Institute of Genomics, Chinese Academy of Sciences (GSA: CRA011142) that are publicly accessible at https://ngdc.cncb.ac.cn/gsa^67,68^.
