# Supplementary figures and images for: Association analysis of agronomic traits and construction of genetic networks by resequencing of 306 sugar beet (Beta vulgaris L.) lines
Source: Sci Rep. 2023 Sep 18;13:15422. doi: 10.1038/s41598-023-42182-2 (PMC10507079; doi:10.1038/s41598-023-42182-2)

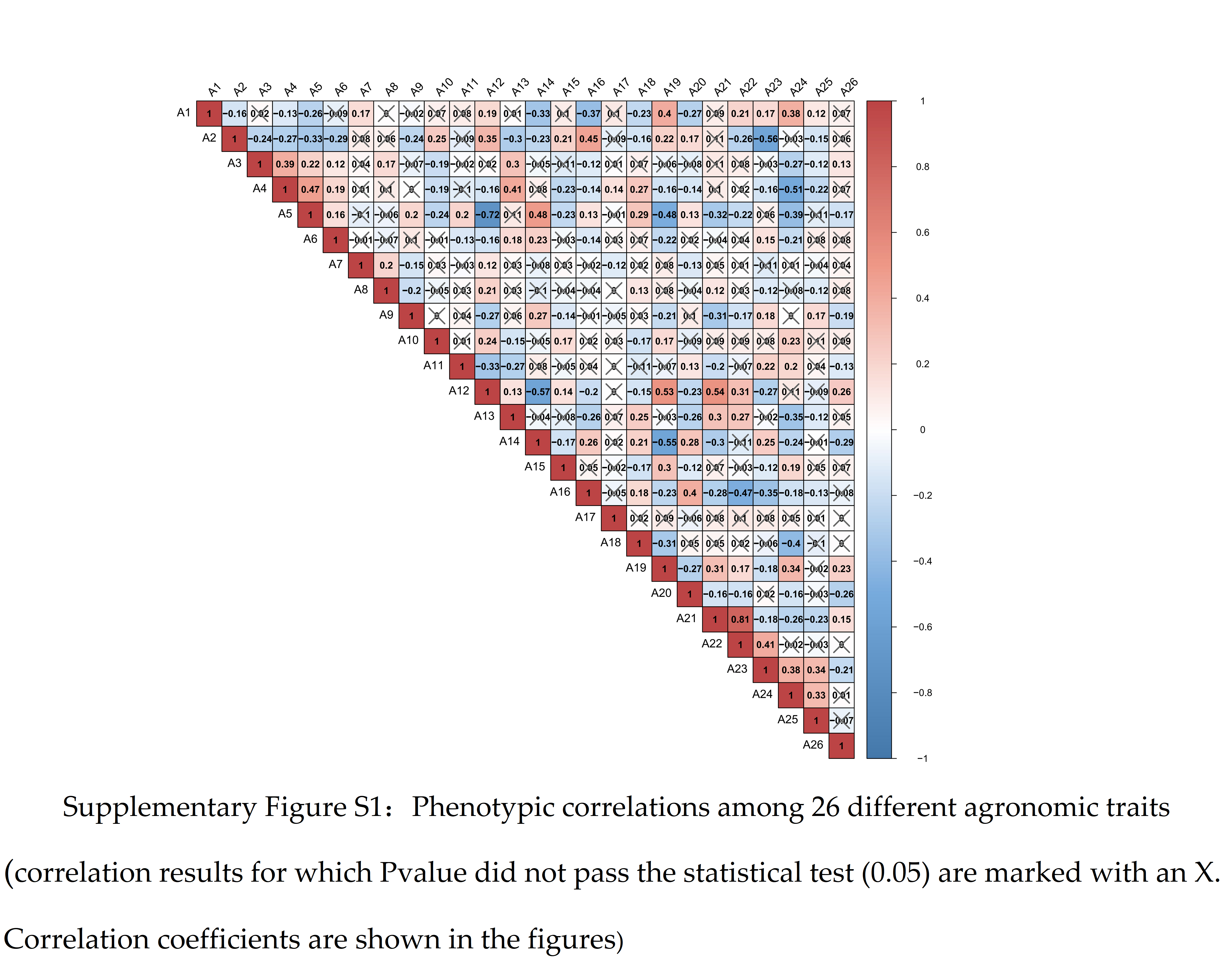

Supplement: Supplementary file 1 — Supplementary Information. [file 41598_2023_42182_MOESM1_ESM.zip › Supplementary/S1-27/S1.jpg]

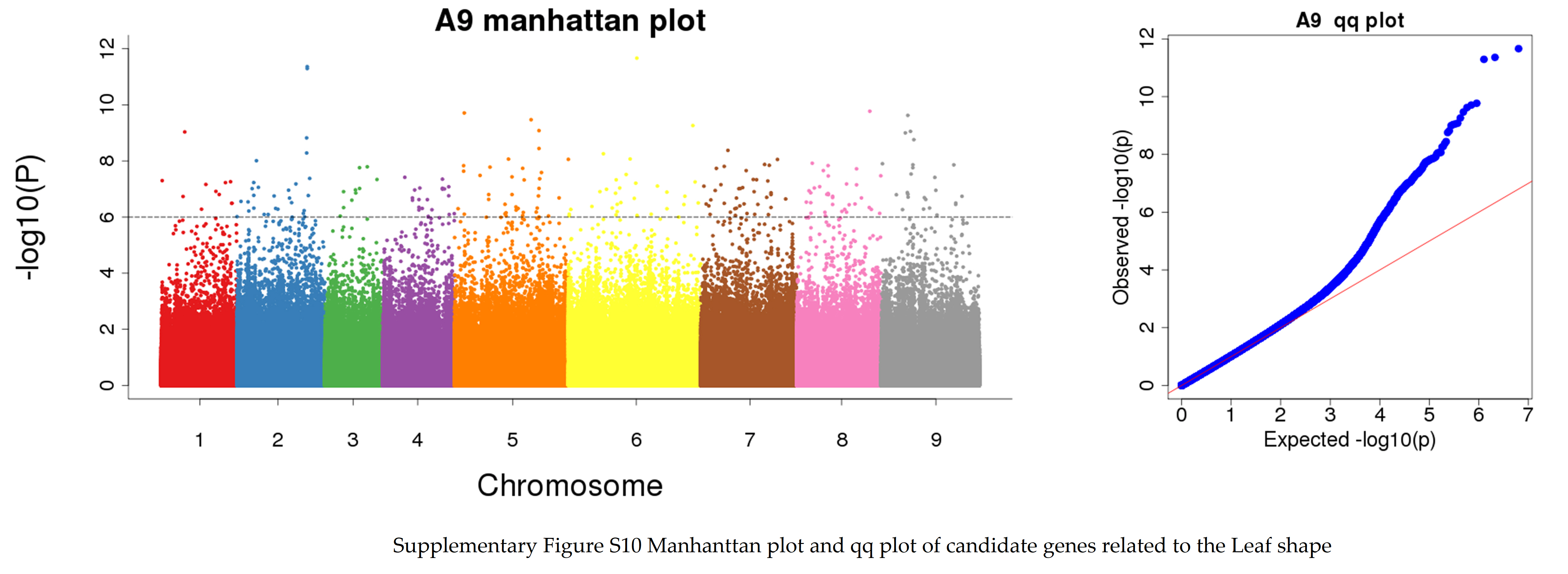

Supplement: Supplementary file 1 — Supplementary Information. [file 41598_2023_42182_MOESM1_ESM.zip › Supplementary/S1-27/S10.jpg]

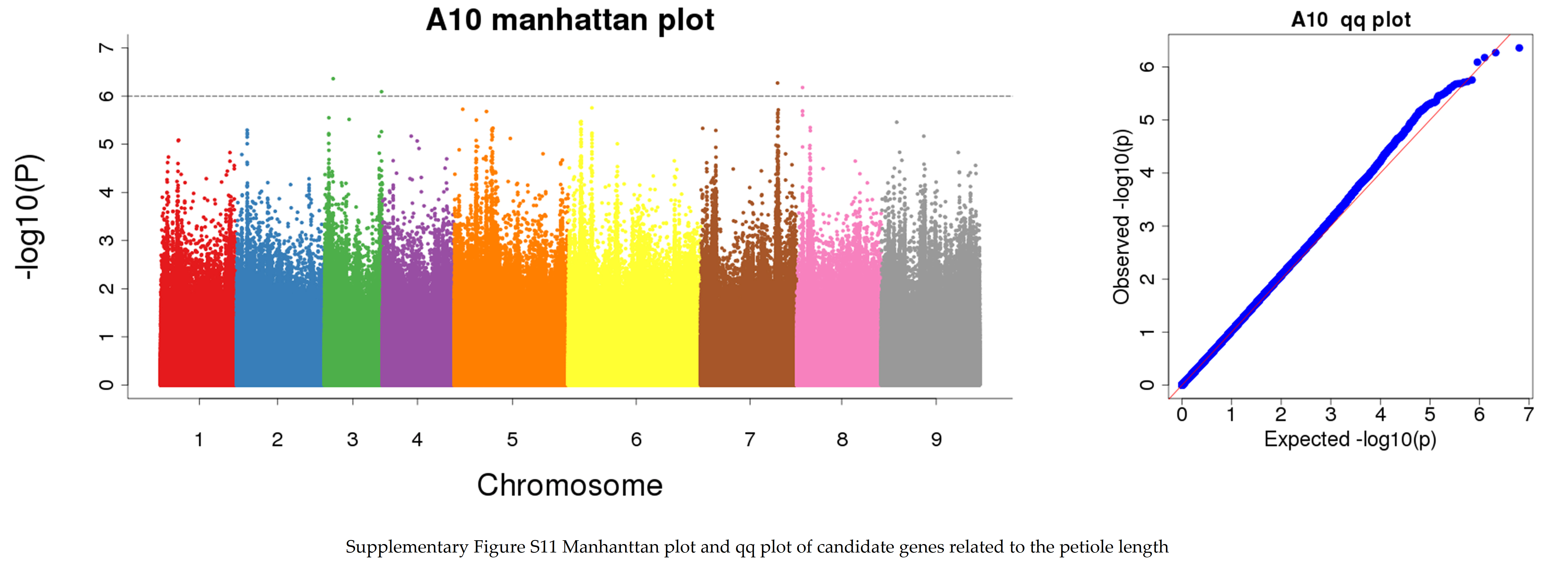

Supplement: Supplementary file 1 — Supplementary Information. [file 41598_2023_42182_MOESM1_ESM.zip › Supplementary/S1-27/S11.jpg]

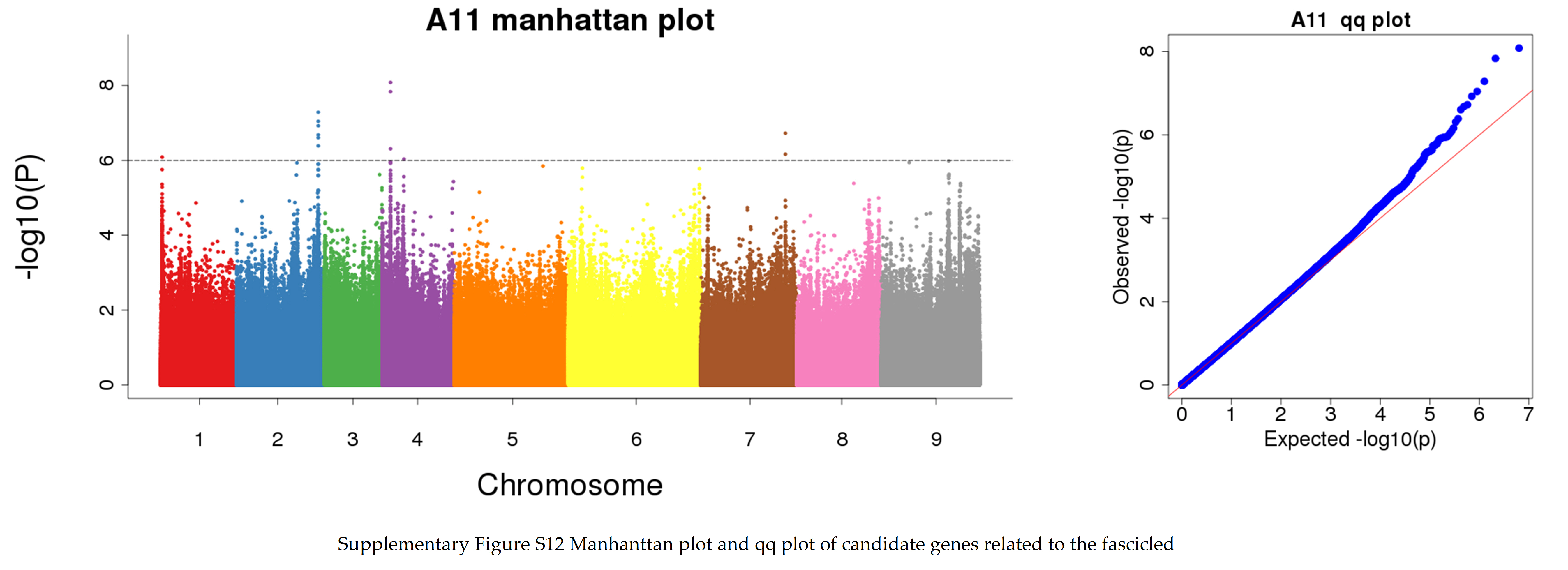

Supplement: Supplementary file 1 — Supplementary Information. [file 41598_2023_42182_MOESM1_ESM.zip › Supplementary/S1-27/S12.jpg]

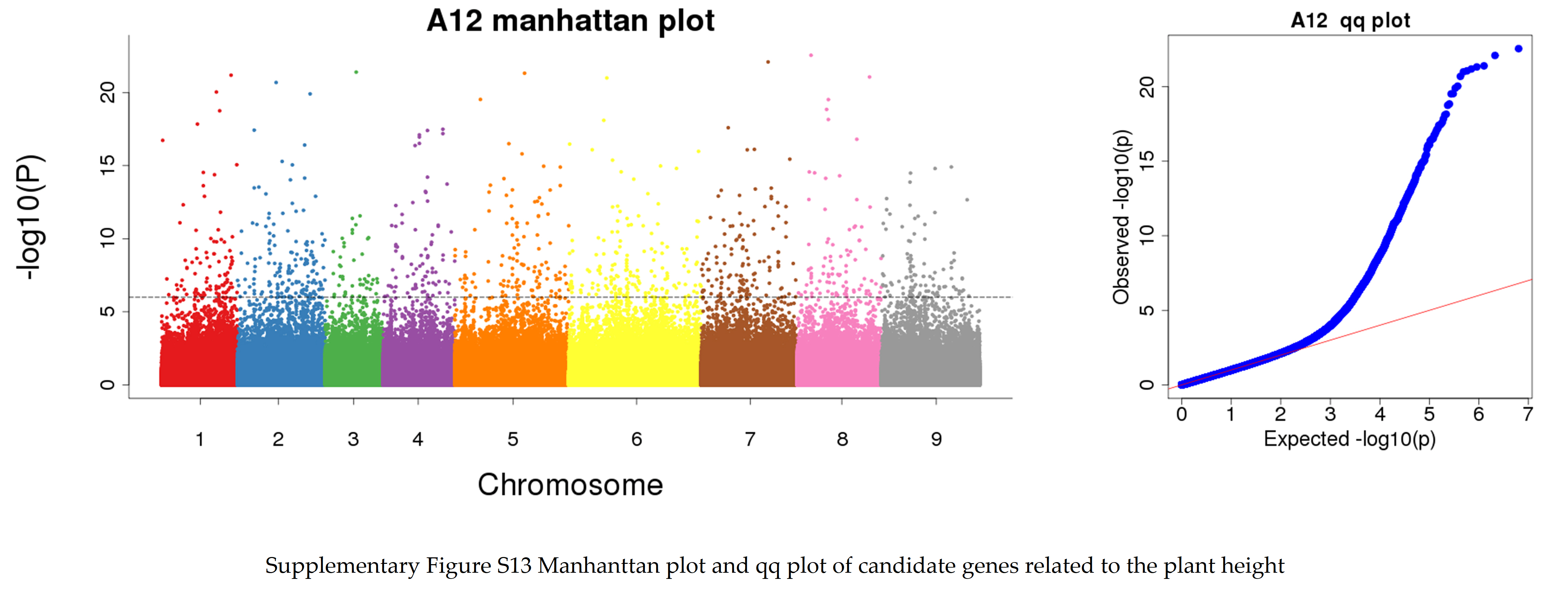

Supplement: Supplementary file 1 — Supplementary Information. [file 41598_2023_42182_MOESM1_ESM.zip › Supplementary/S1-27/S13.jpg]

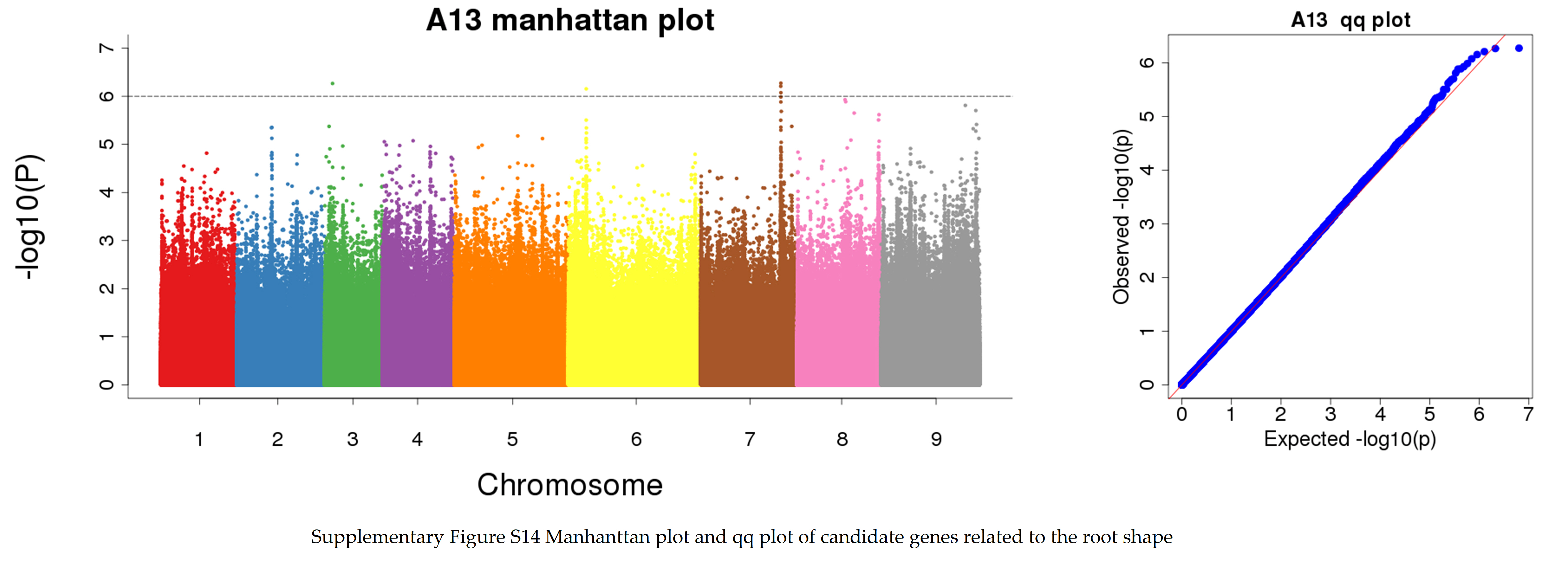

Supplement: Supplementary file 1 — Supplementary Information. [file 41598_2023_42182_MOESM1_ESM.zip › Supplementary/S1-27/S14.jpg]

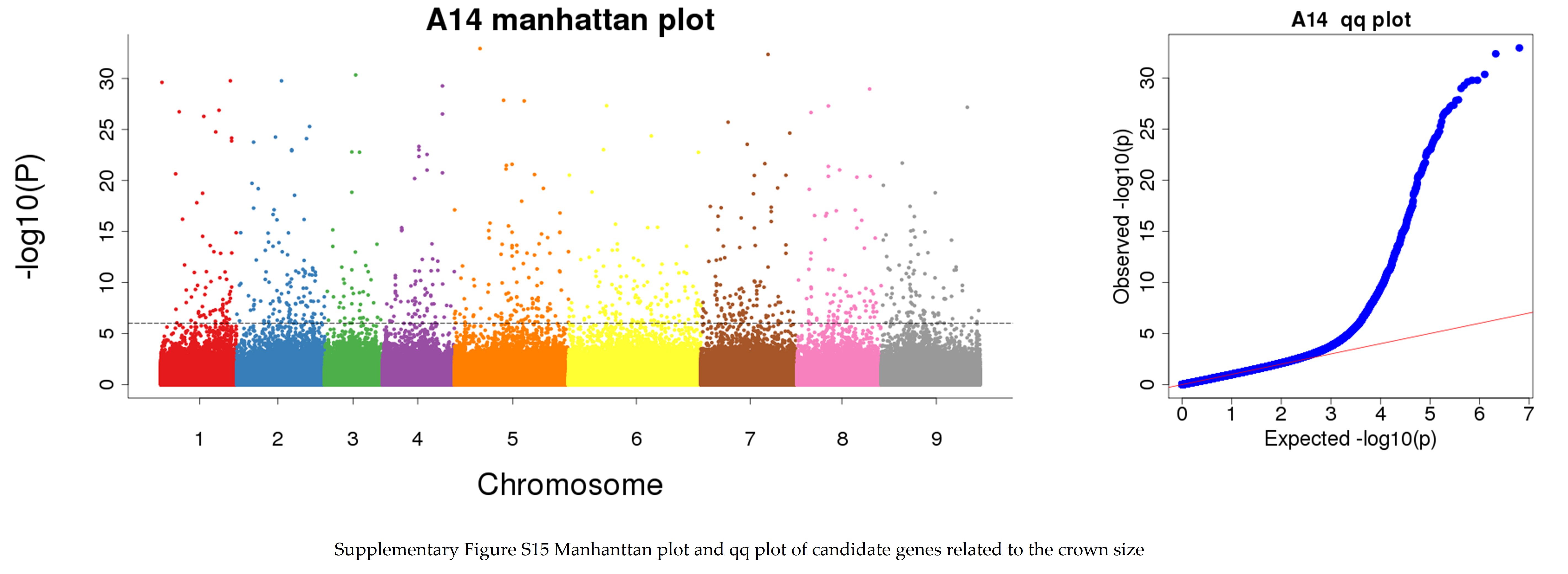

Supplement: Supplementary file 1 — Supplementary Information. [file 41598_2023_42182_MOESM1_ESM.zip › Supplementary/S1-27/S15.jpg]

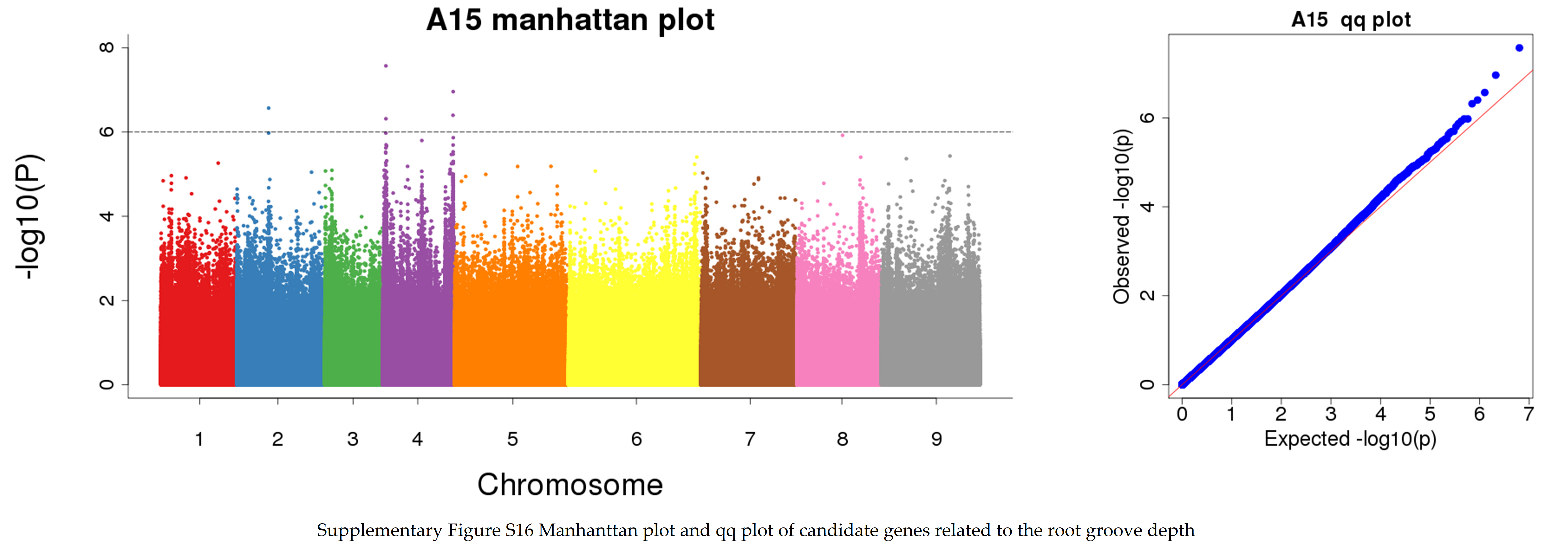

Supplement: Supplementary file 1 — Supplementary Information. [file 41598_2023_42182_MOESM1_ESM.zip › Supplementary/S1-27/S16.jpg]

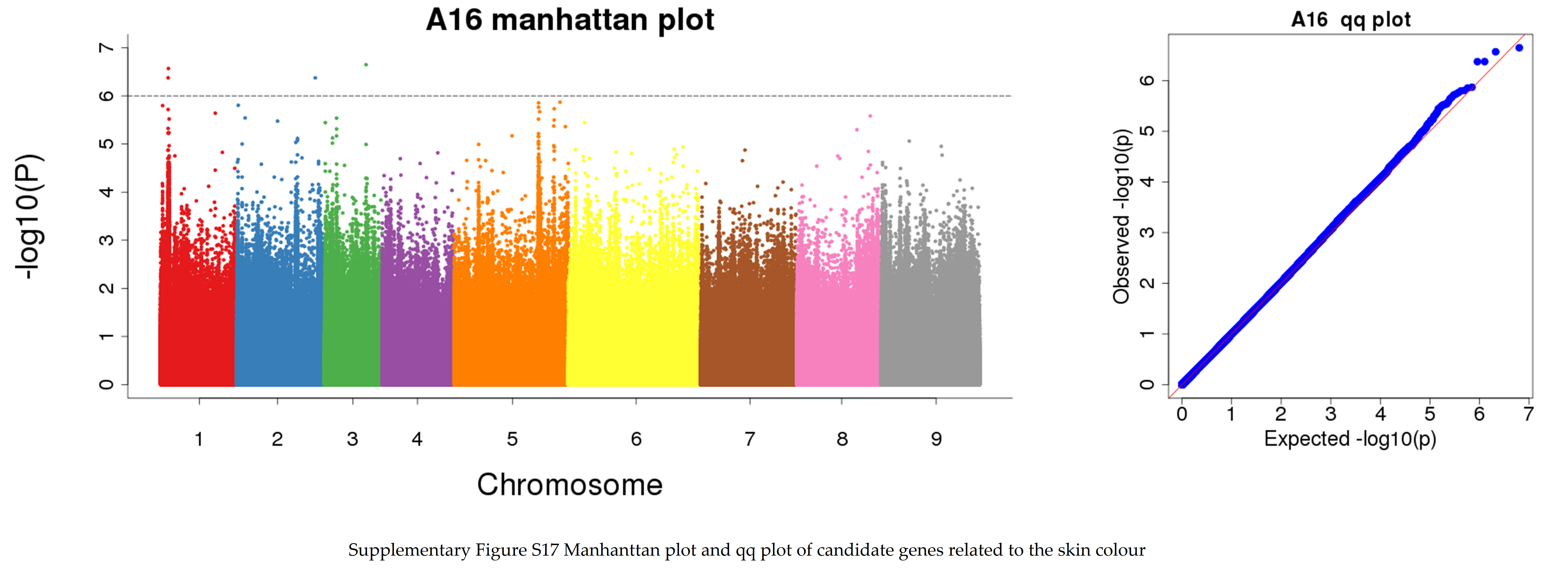

Supplement: Supplementary file 1 — Supplementary Information. [file 41598_2023_42182_MOESM1_ESM.zip › Supplementary/S1-27/S17.jpg]

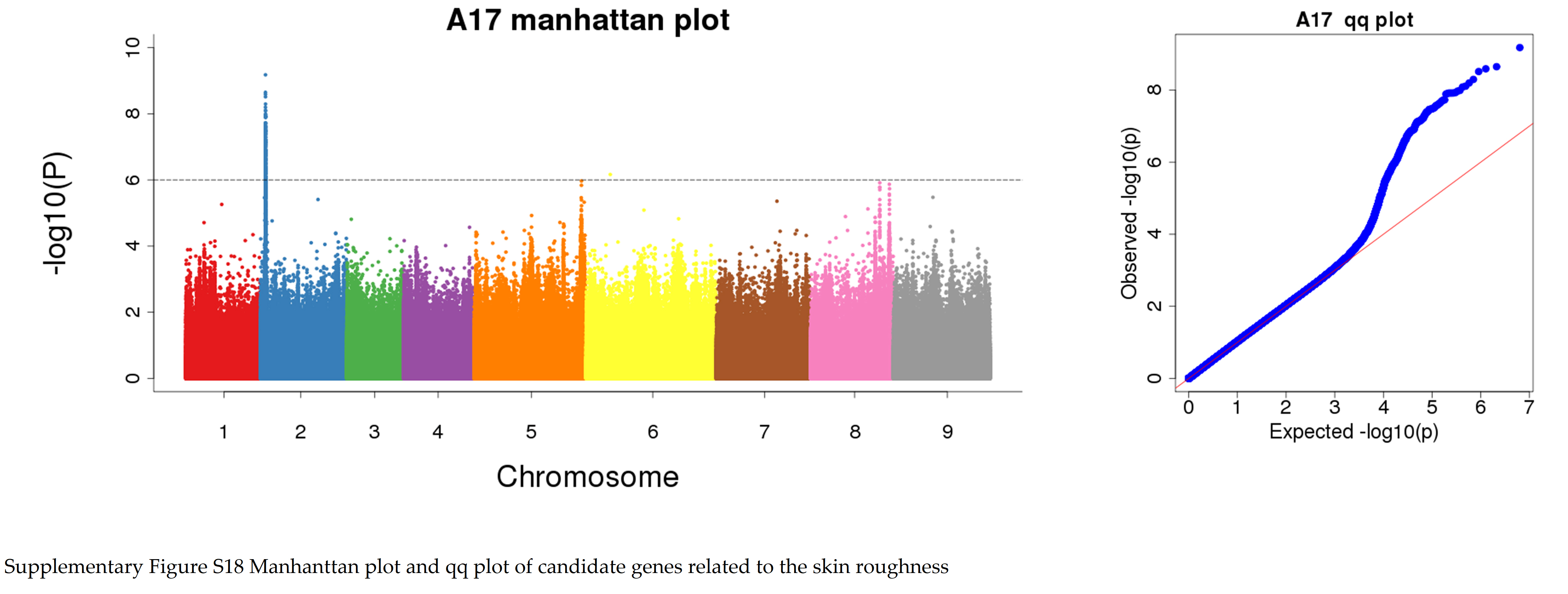

Supplement: Supplementary file 1 — Supplementary Information. [file 41598_2023_42182_MOESM1_ESM.zip › Supplementary/S1-27/S18.jpg]

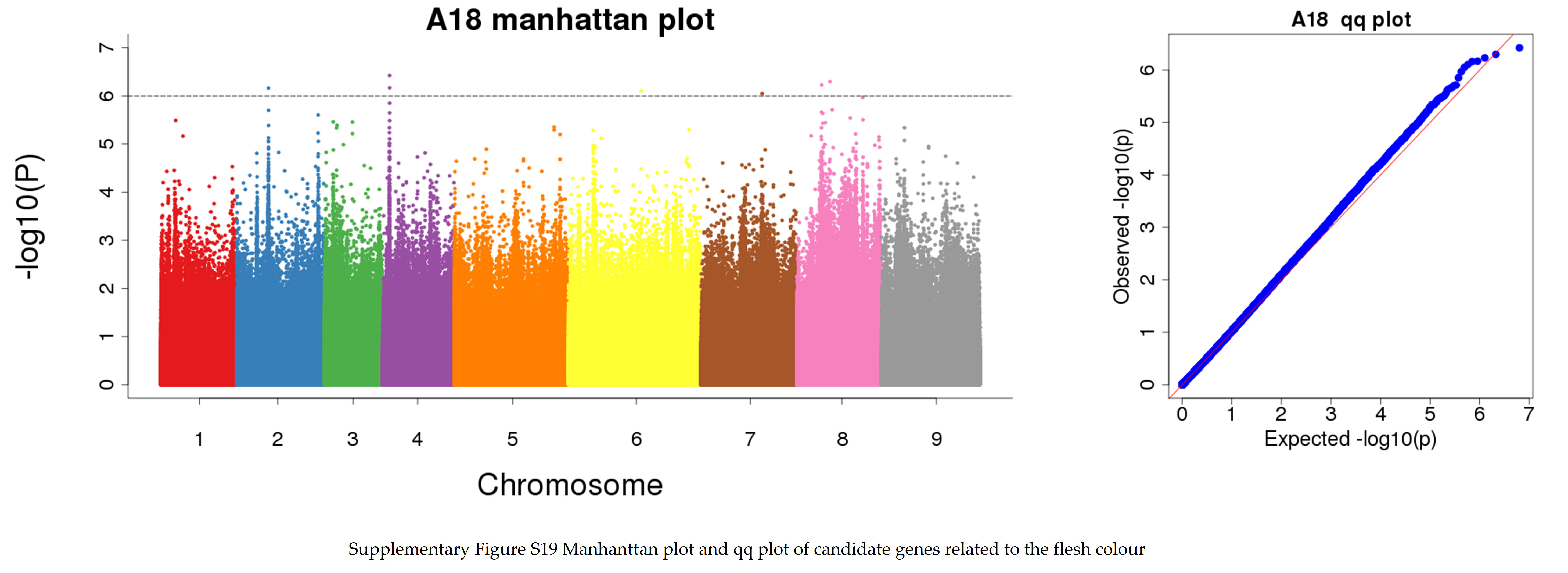

Supplement: Supplementary file 1 — Supplementary Information. [file 41598_2023_42182_MOESM1_ESM.zip › Supplementary/S1-27/S19.jpg]

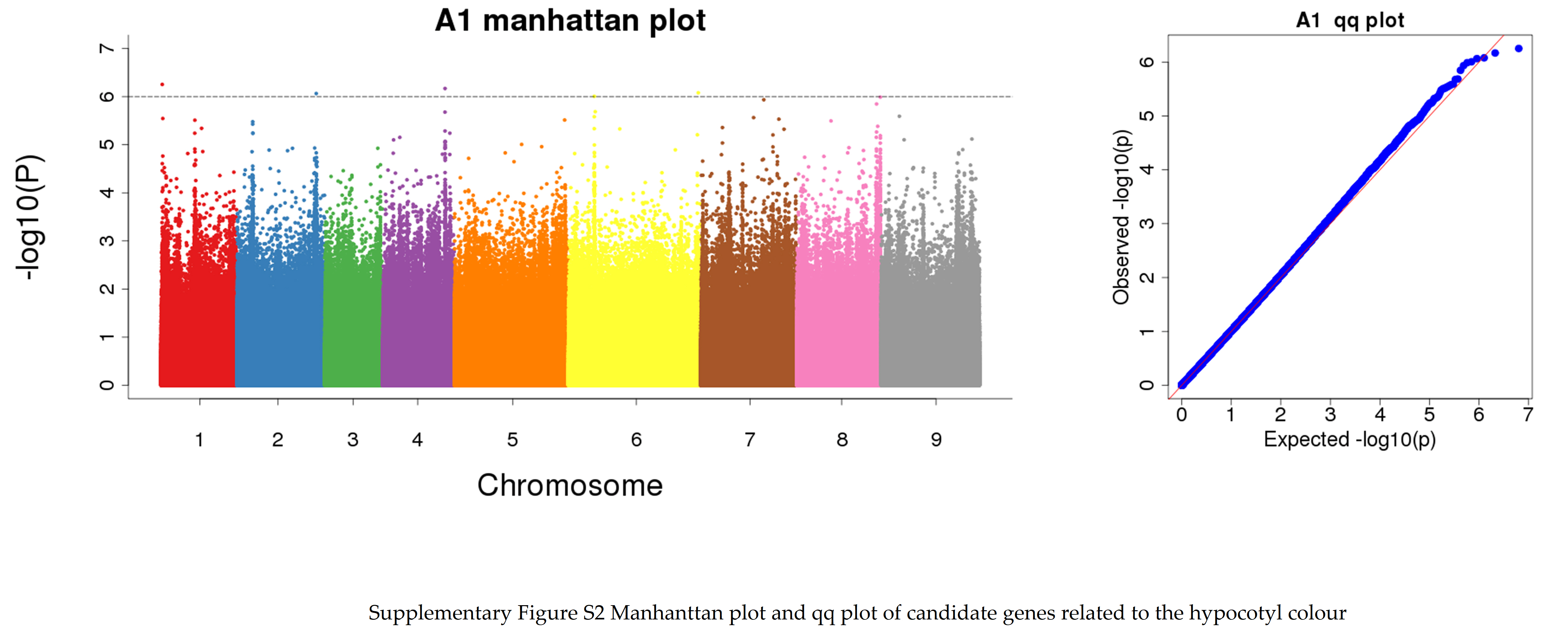

Supplement: Supplementary file 1 — Supplementary Information. [file 41598_2023_42182_MOESM1_ESM.zip › Supplementary/S1-27/S2.jpg]

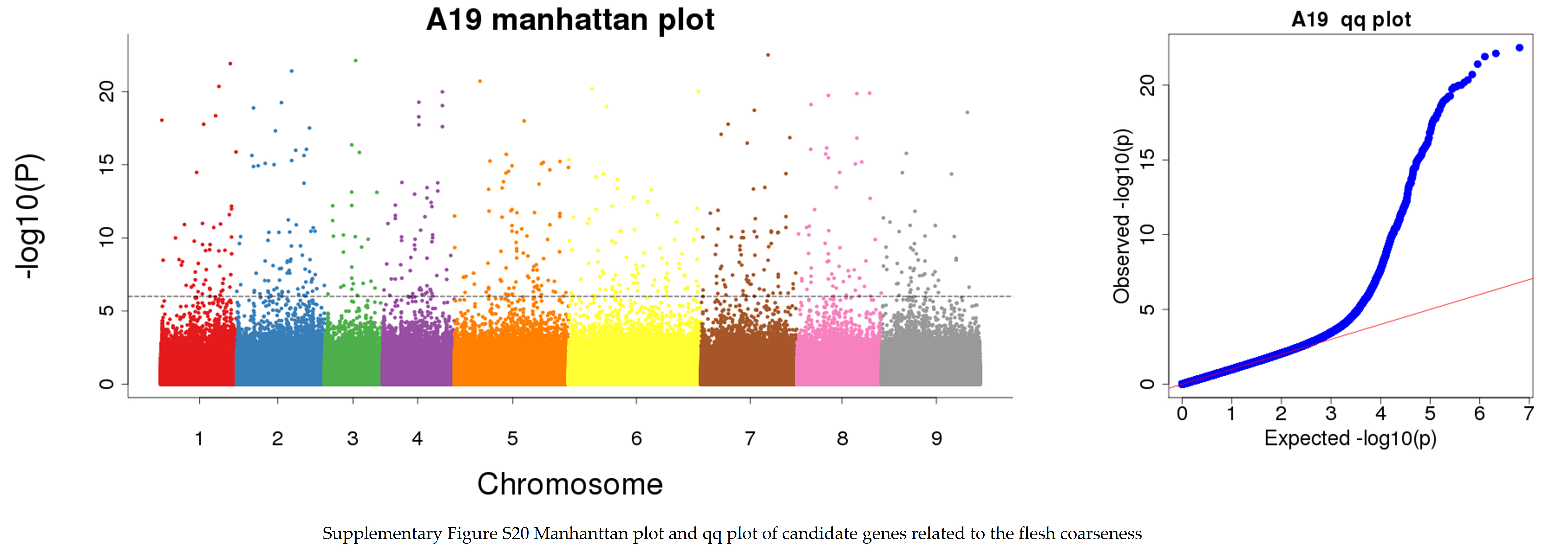

Supplement: Supplementary file 1 — Supplementary Information. [file 41598_2023_42182_MOESM1_ESM.zip › Supplementary/S1-27/S20.jpg]

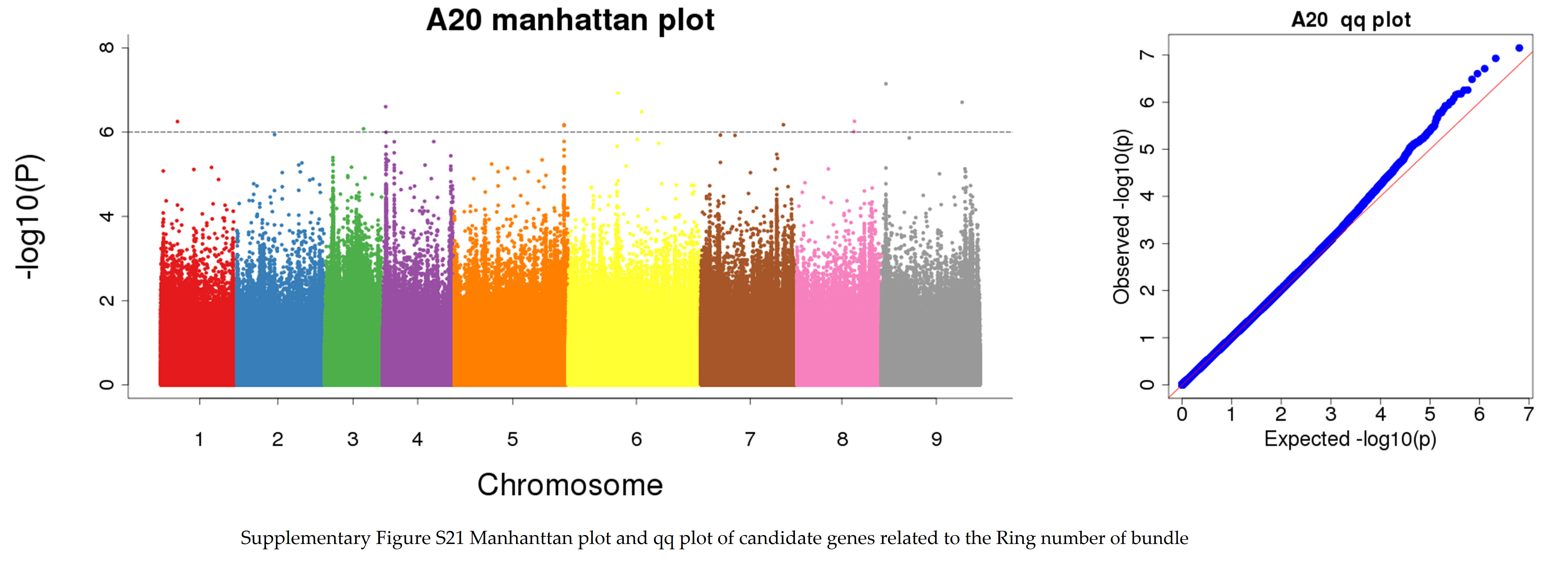

Supplement: Supplementary file 1 — Supplementary Information. [file 41598_2023_42182_MOESM1_ESM.zip › Supplementary/S1-27/S21.jpg]

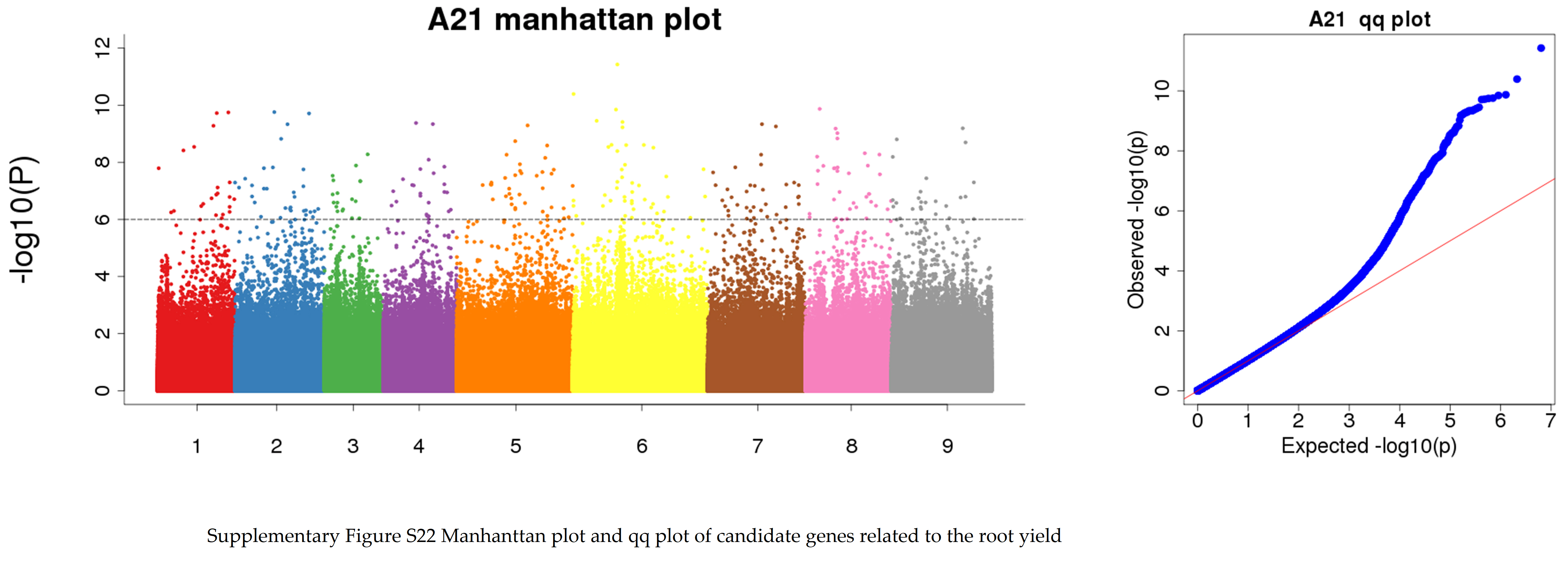

Supplement: Supplementary file 1 — Supplementary Information. [file 41598_2023_42182_MOESM1_ESM.zip › Supplementary/S1-27/S22.jpg]

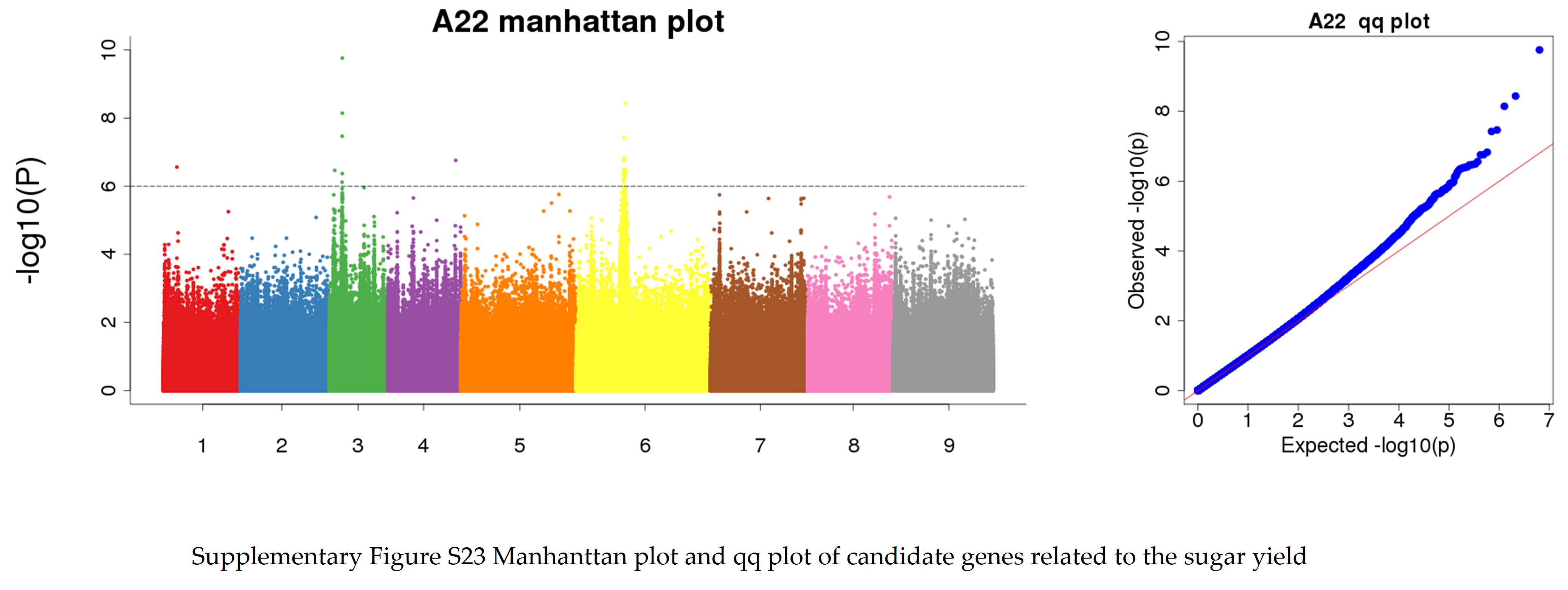

Supplement: Supplementary file 1 — Supplementary Information. [file 41598_2023_42182_MOESM1_ESM.zip › Supplementary/S1-27/S23.jpg]

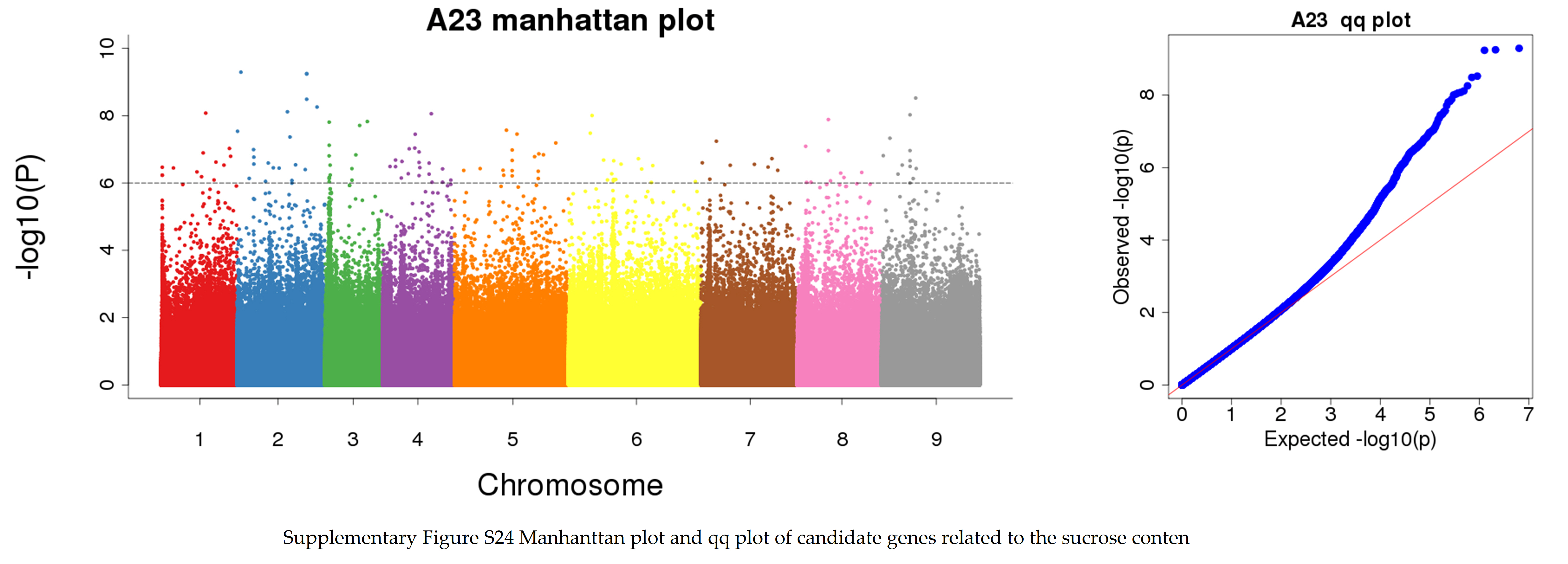

Supplement: Supplementary file 1 — Supplementary Information. [file 41598_2023_42182_MOESM1_ESM.zip › Supplementary/S1-27/S24.jpg]

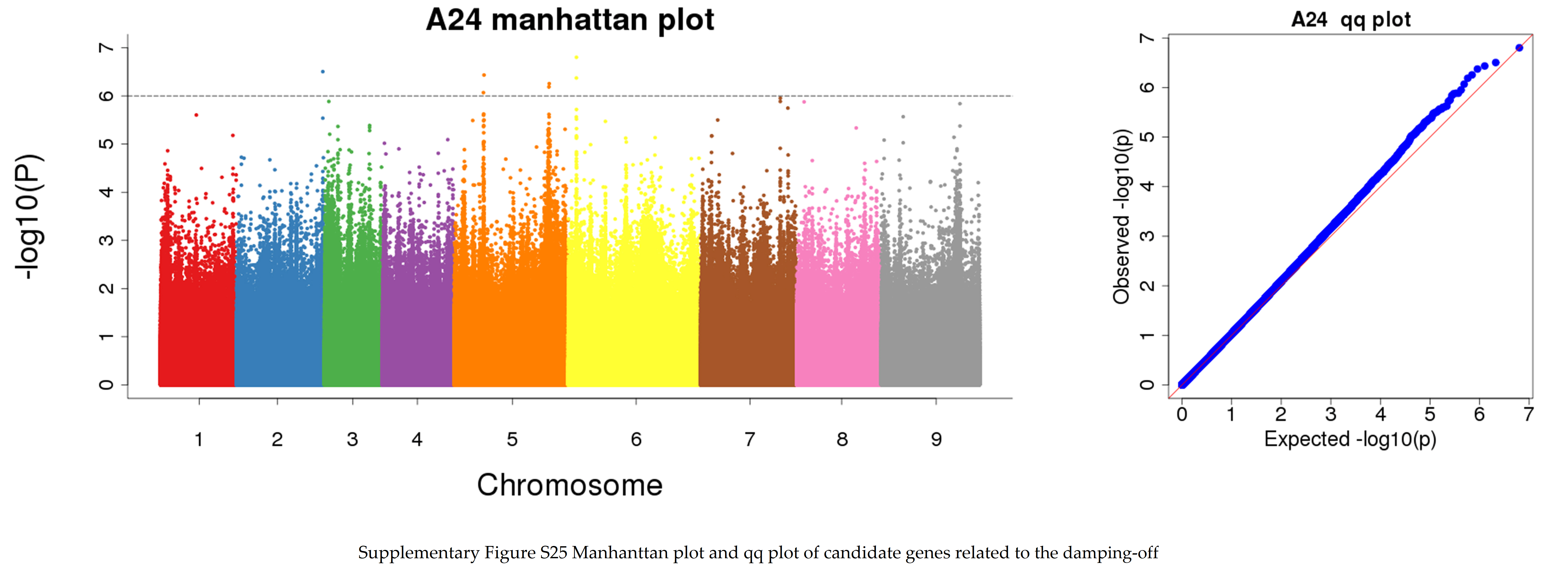

Supplement: Supplementary file 1 — Supplementary Information. [file 41598_2023_42182_MOESM1_ESM.zip › Supplementary/S1-27/S25.jpg]

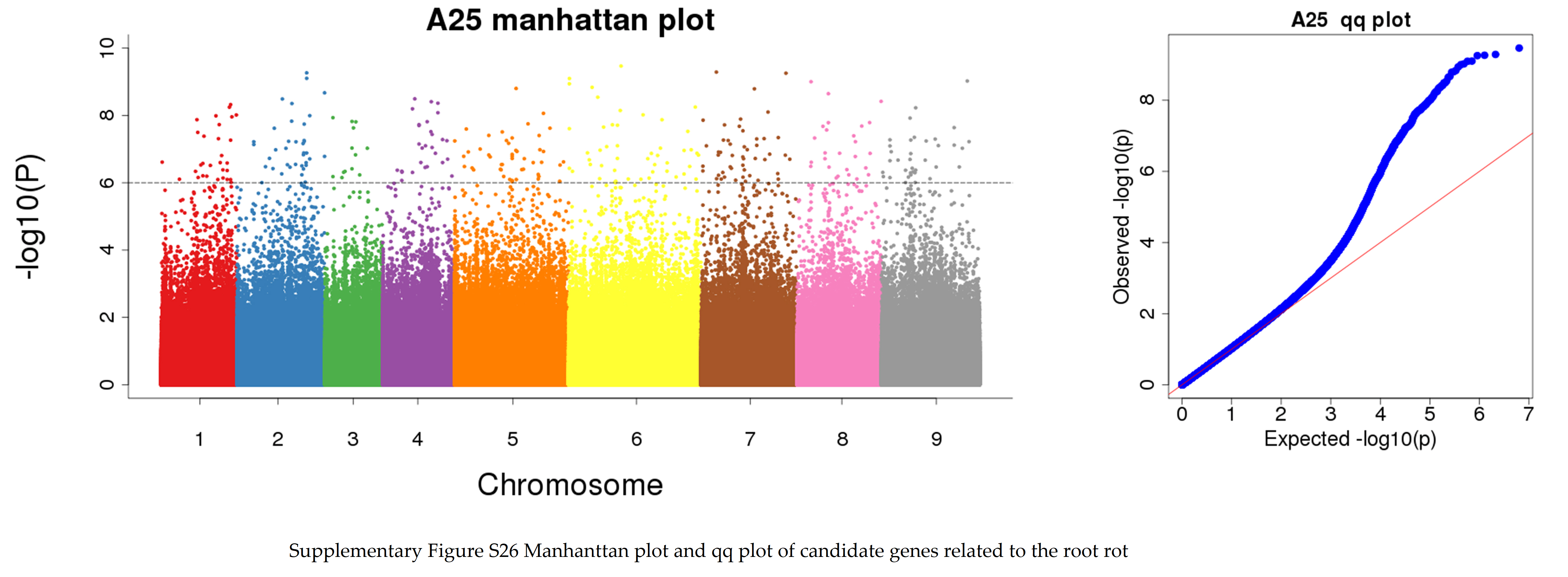

Supplement: Supplementary file 1 — Supplementary Information. [file 41598_2023_42182_MOESM1_ESM.zip › Supplementary/S1-27/S26.jpg]

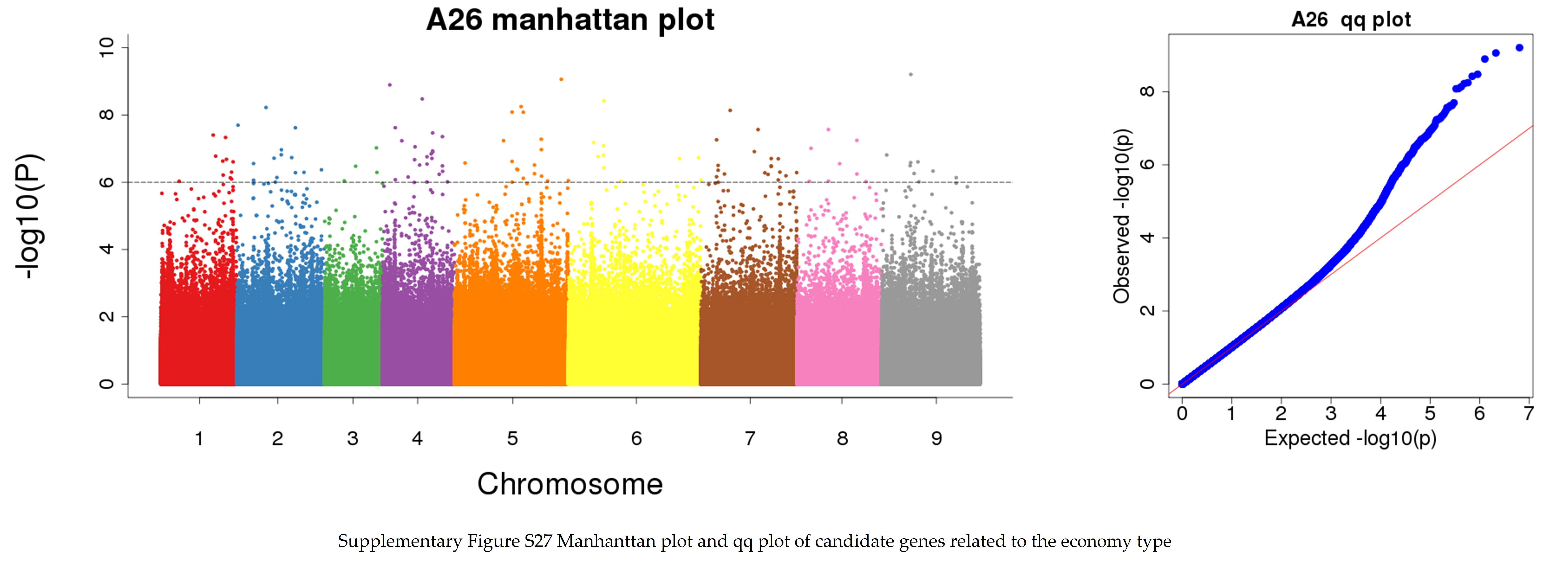

Supplement: Supplementary file 1 — Supplementary Information. [file 41598_2023_42182_MOESM1_ESM.zip › Supplementary/S1-27/S27.jpg]

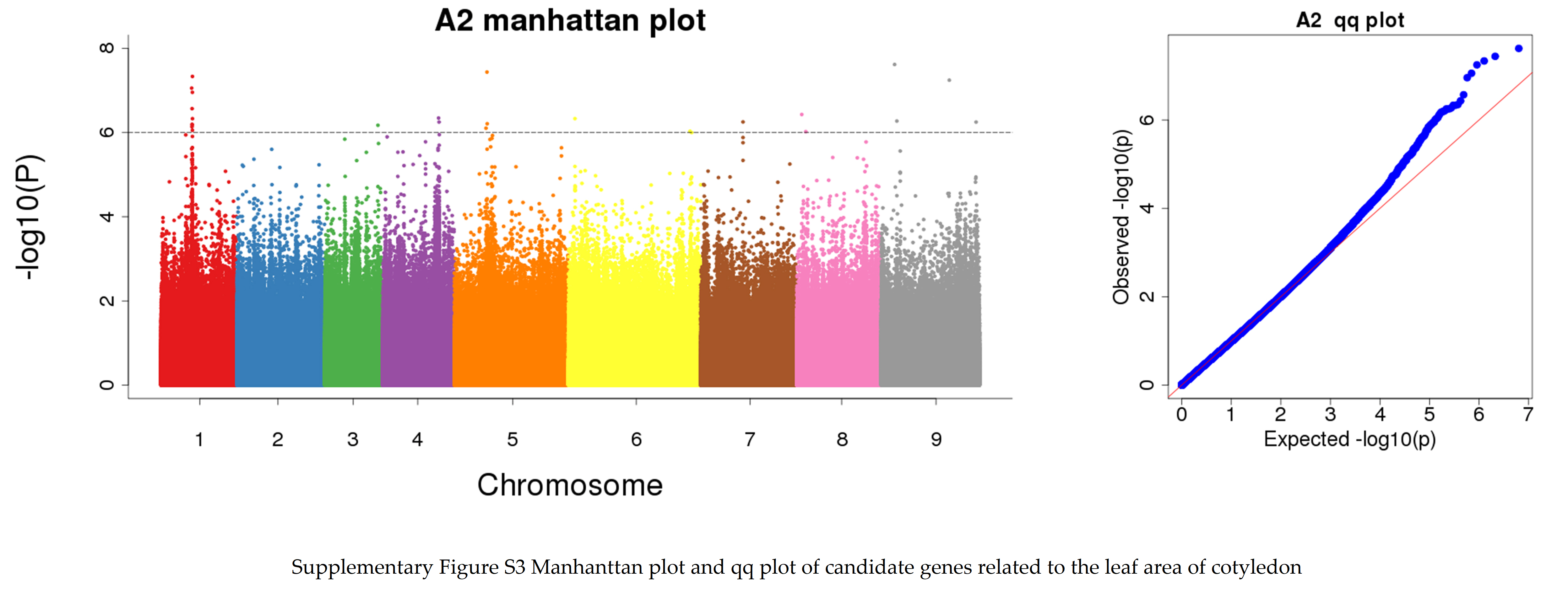

Supplement: Supplementary file 1 — Supplementary Information. [file 41598_2023_42182_MOESM1_ESM.zip › Supplementary/S1-27/S3.jpg]

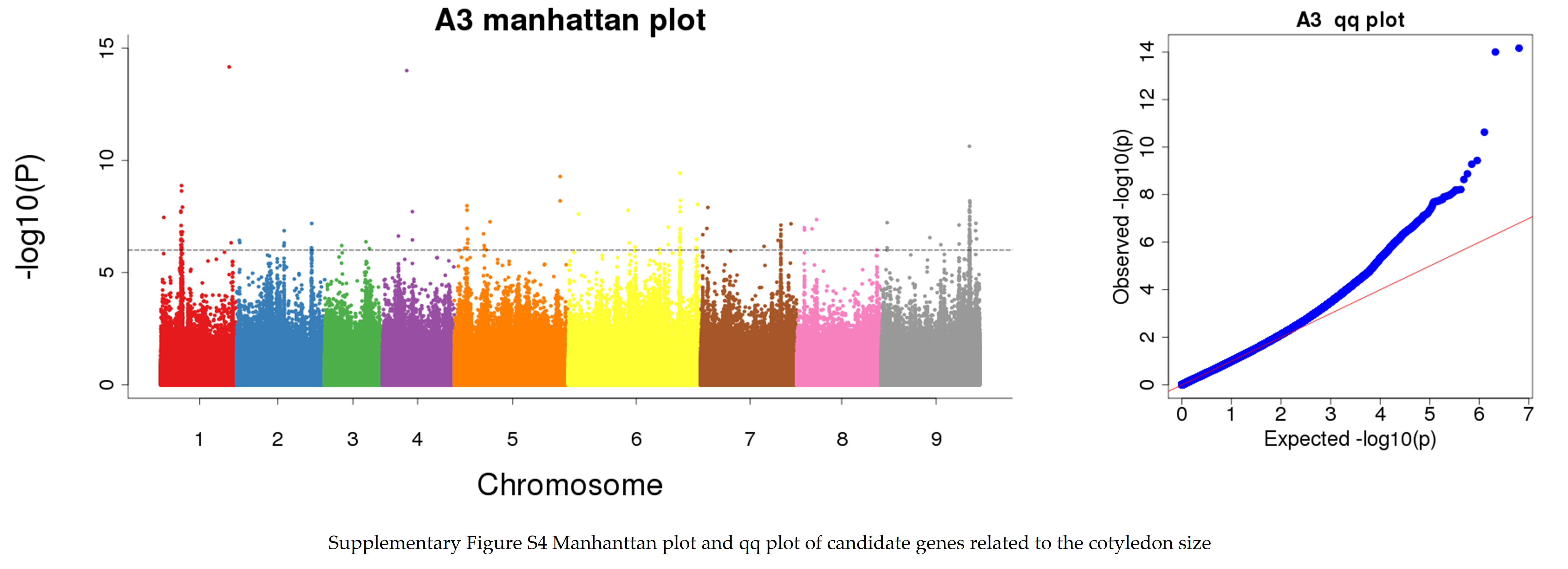

Supplement: Supplementary file 1 — Supplementary Information. [file 41598_2023_42182_MOESM1_ESM.zip › Supplementary/S1-27/S4.jpg]

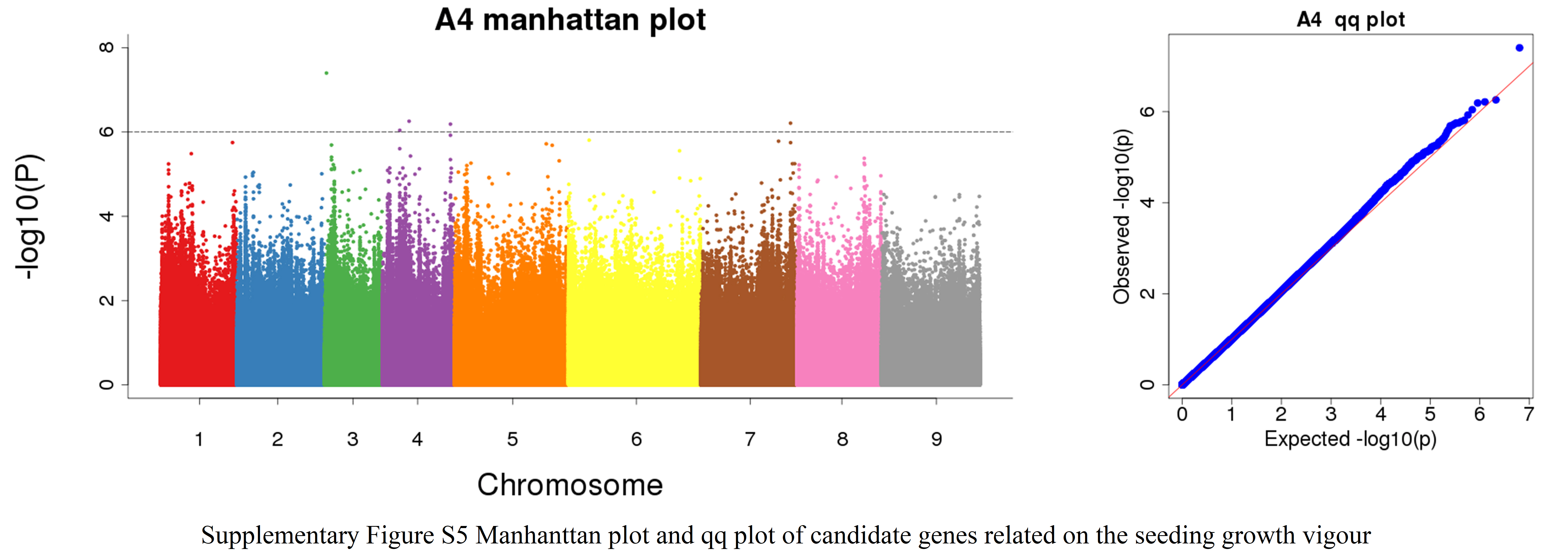

Supplement: Supplementary file 1 — Supplementary Information. [file 41598_2023_42182_MOESM1_ESM.zip › Supplementary/S1-27/S5.jpg]

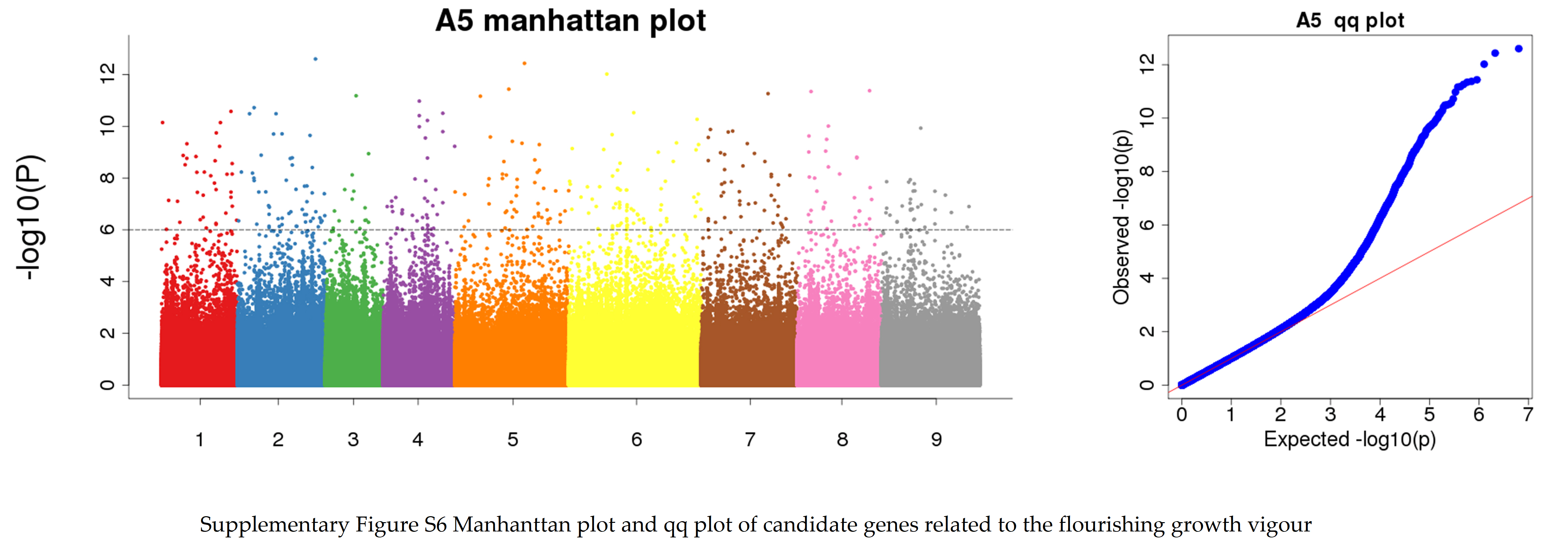

Supplement: Supplementary file 1 — Supplementary Information. [file 41598_2023_42182_MOESM1_ESM.zip › Supplementary/S1-27/S6.jpg]

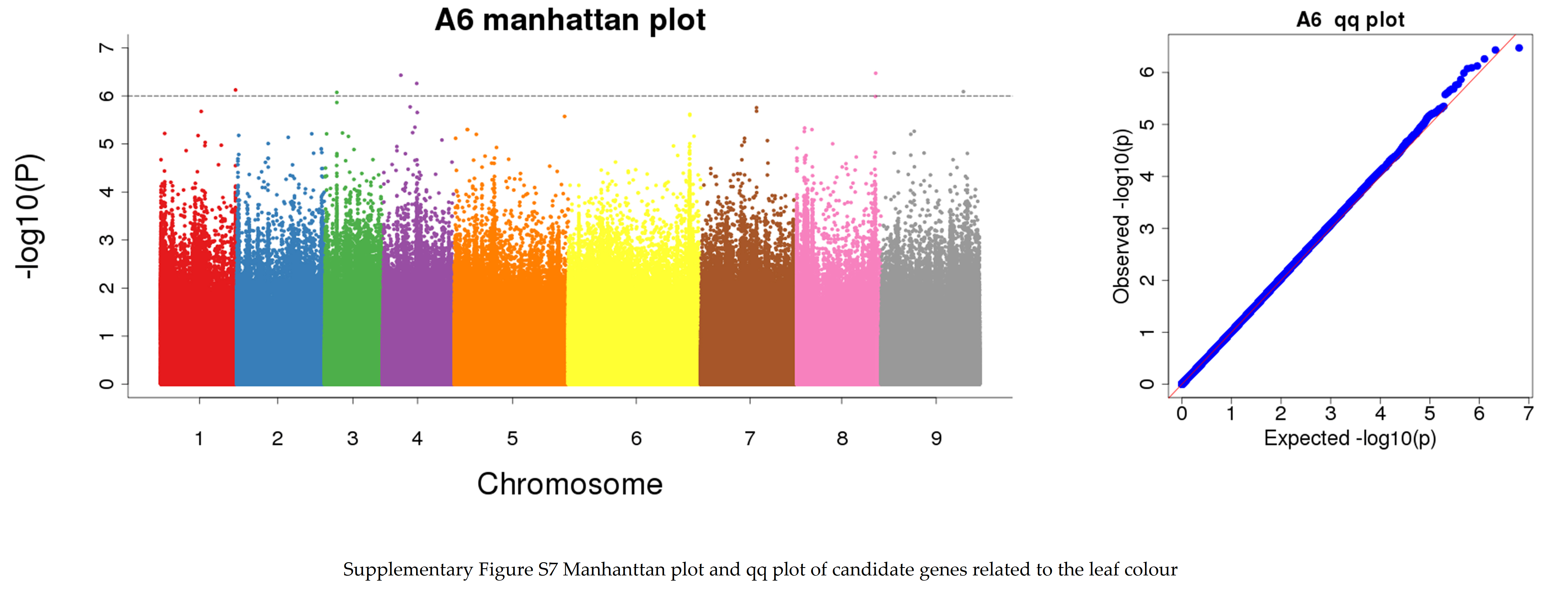

Supplement: Supplementary file 1 — Supplementary Information. [file 41598_2023_42182_MOESM1_ESM.zip › Supplementary/S1-27/S7.jpg]

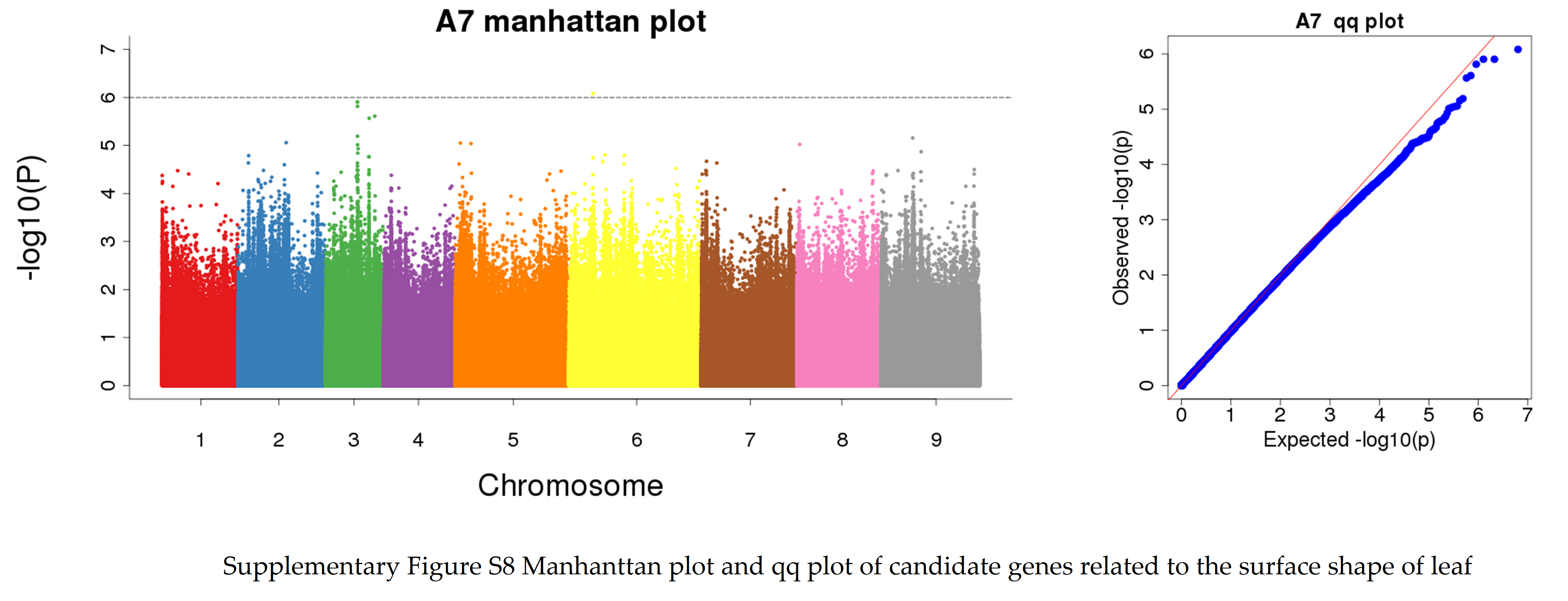

Supplement: Supplementary file 1 — Supplementary Information. [file 41598_2023_42182_MOESM1_ESM.zip › Supplementary/S1-27/S8.jpg]

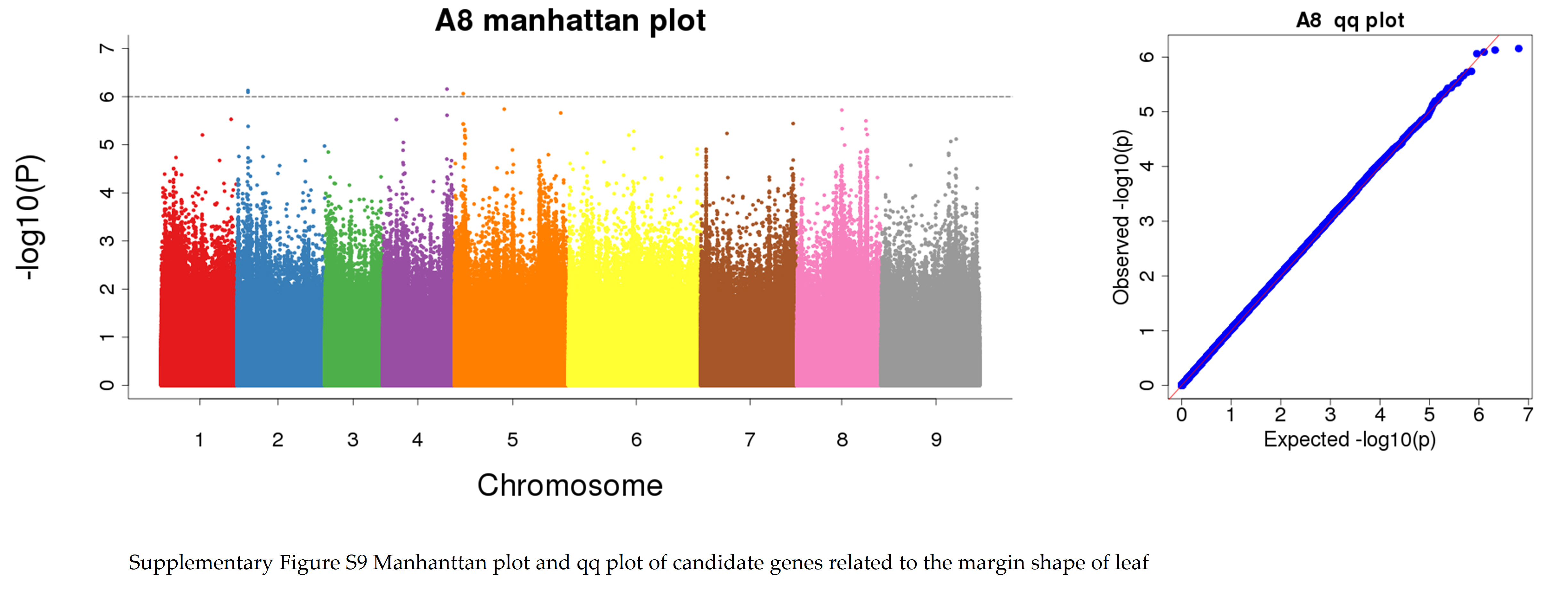

Supplement: Supplementary file 1 — Supplementary Information. [file 41598_2023_42182_MOESM1_ESM.zip › Supplementary/S1-27/S9.jpg]
